# Supplementary material for: Mechanical loading of intraluminal pressure mediates wound angiogenesis by regulating the TOCA family of F-BAR proteins
Source: Nat Commun. 2022 May 12;13:2594. doi: 10.1038/s41467-022-30197-8 (PMC9098626; doi:10.1038/s41467-022-30197-8)
Supplement: Supplementary file 1 — Supplementary Information [file 41467_2022_30197_MOESM1_ESM.pdf]

SUPPLEMENTARY INFORMATION

Mechanical loading of intraluminal pressure mediates wound angiogenesis by regulating the TOCA family of F-BAR proteins

Shinya Yuge, Koichi Nishiyama, Yuichiro Arima, Yasuyuki Hanada,
Eri Oguri-Nakamura, Sanshiro Hanada, Tomohiro Ishii, Yuki Wakayama,
Urara Hasegawa, Kazuya Tsujita, Ryuji Yokokawa, Takashi Miura, Toshiki Itoh,
Kenichi Tsujita, Naoki Mochizuki, Shigetomo Fukuhara

INVENTORY OF SUPPLEMENTARY INFORMATION

Supplementary Figures 1-21

Supplementary Tables 1-4

Supplemental Figures

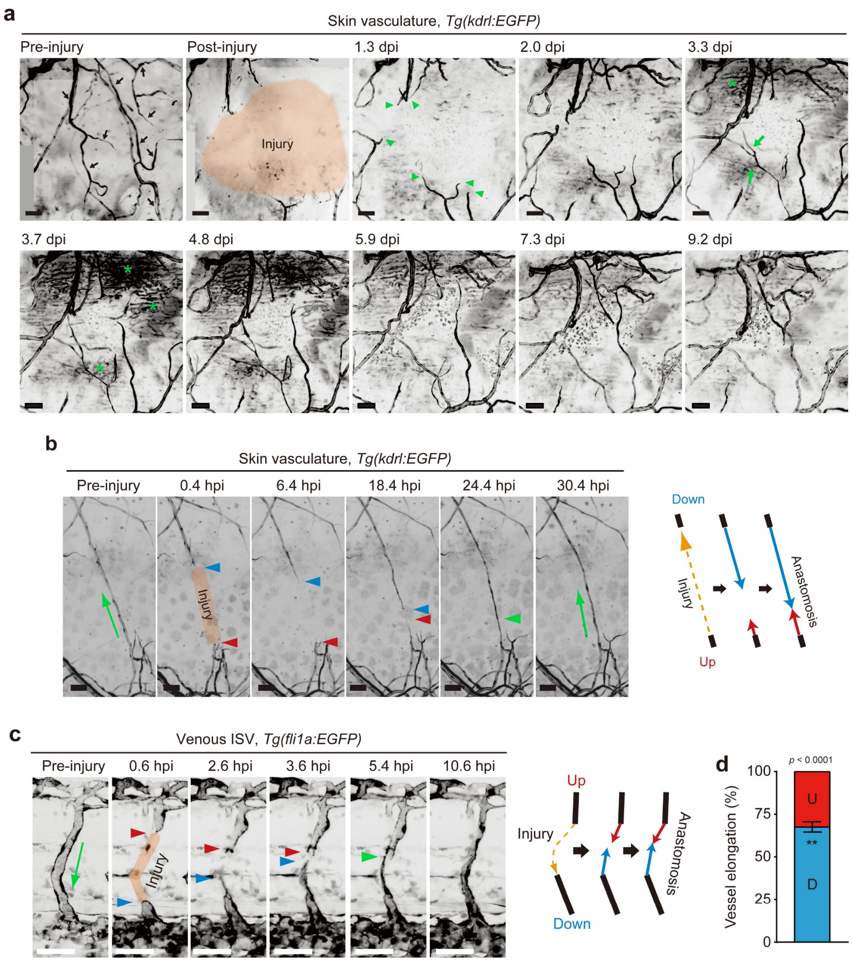

Yuge, Nishiyama et al. Supplementary Figure 1

Supplementary Fig. 1 Preferential elongation of injured blood vessels downstream from blood flow during wound angiogenesis. a Time-lapse images of angiogenesis in the injured skin of the *Tg(kdrl:EGFP)* adult zebrafish. Confocal z-projection images cutaneous vasculature before (Pre-injury) and after (Post-injury) injury and at the elapsed time following the injury (dpi: days post-injury). Cutaneous vessel networks in adult zebrafish consist of not only blood vessels but also vessels not containing circulating erythrocytes (black arrows) {Noishiki, 2019 #1935}. In this study, we focused on the blood vessels. Orange (Post-injury), injured area; green arrowheads (1.3 dpi), elongating severed blood vessels; green arrows (3.3 dpi), blood vessels sprouting from pre-existing vessels; asterisk (3.3 and 3.7 dpi), vascular plexus translocated from the muscle layer. See also Supplementary Movie 1. **b** Time-lapse images of repair process of an injured single capillary in the skin of the *Tg(kdrl:EGFP)* adult zebrafish. Confocal z-projection images before (Pre-injury) and after (Post-injury) injury and at

the elapsed time following the injury (hpi: hours post-injury). In contrast to the experiments described in Fig. 1b, only a small portion of the capillary (approximately 260 µm in length) was injured in this experiment. Repair process of the injured capillary is depicted on the right. Orange, injured area; red and blue arrowheads, leading edges of the injured vessels at sites upstream and downstream from the blood flow, respectively; green arrowhead, anastomotic site of the injured vessels; green arrow, direction of blood flow. Note that injured blood vessels mainly elongate downstream, not upstream, from the blood flow, as depicted on the right. See also Supplementary Movie 4. **c** Time-lapse images of repair process of the injured venous ISV in the *Tg(fli1a:EGFP)* zebrafish larva at 3 dpf. Confocal z-projection images before injury (Pre-injury) and at the elapsed time following the injury (hpi) are shown as in **b**. Lateral view, anterior to the left. Note that the injured venous ISV located downstream from the blood flow preferentially elongated as depicted on the right. See also Supplementary Movie 6. **d** Elongation (represented as a measurement of length) of the upstream (red) and downstream injured (blue) vessels as observed in **c** are expressed as a percentage of the total amount of elongation. Data are shown as means ± s.e.m. (n = 4 animals). $^{**}p < 0.01$ by two-sided t-test. Source data are provided as a Source Data file. Scale bars: 50 µm (**a**, **b**, **c**).

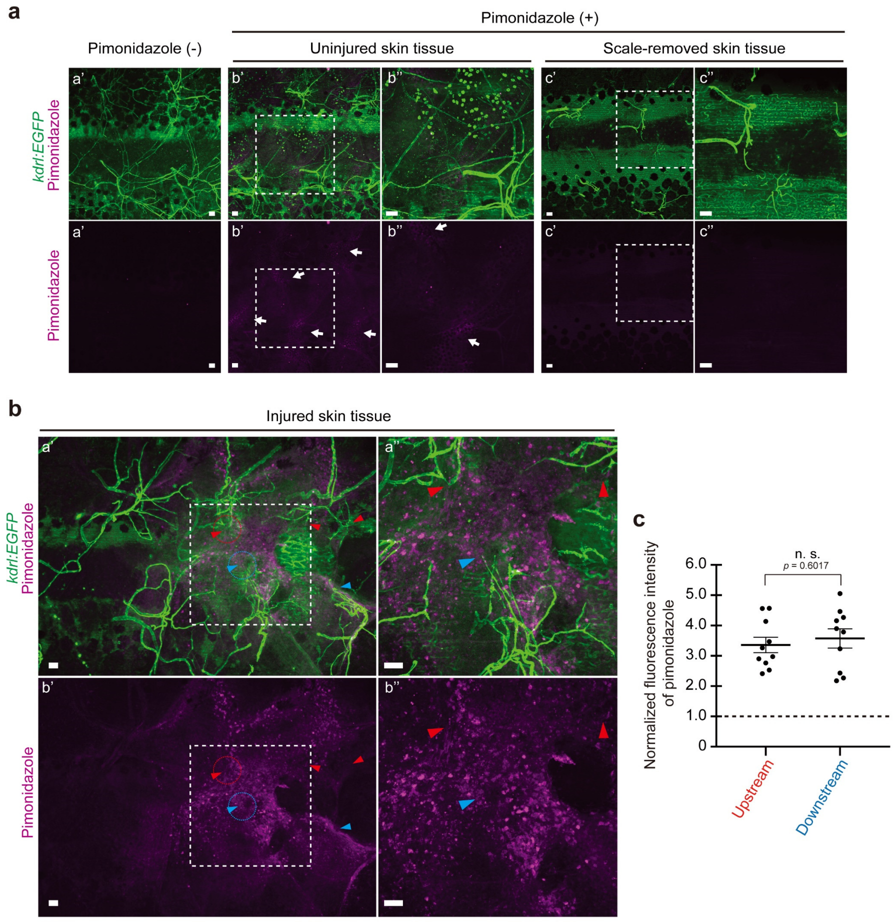

Yuge, Nishiyama et al. Supplementary Figure 2

Supplementary Fig. 2 No significant difference in hypoxic states between tissues surrounding downstream and upstream injured vessels. a Detection of hypoxia in the skin of adult zebrafish. Confocal z-projection images of uninjured (a’, b’, b’’) and scale-removed (c’, c’’) skin tissues in the adult *Tg(kdrl:EGFP)* zebrafish intraperitoneally injected without (a’) and with (b’, b’’, c’, c’’) pimonidazole. Upper panel, the merged images of EGFP (green) and pimonidazole (magenta); lower panel, pimonidazole image (magenta). The boxed areas are enlarged on the right. Note that the scales show weak pimonidazole staining (arrows), indicating that the scales of adult zebrafish are moderately hypoxic. **b**, **c** Detection of hypoxia in the injured skin of adult zebrafish. **b** Confocal z-projection images of the injured skin tissue in the adult *Tg(kdrl:EGFP)* zebrafish intraperitoneally injected with pimonidazole are shown as in **a**. Red and blue arrowheads, the ends of injured blood vessels at the sites upstream and downstream from the blood flow, respectively. **c** Quantification of hypoxic states in the areas surrounding the injured upstream and downstream vessels. Fluorescence intensity of pimonidazole staining in the circular regions with a diameter of 30 μm around the ends of upstream and downstream injured vessels as indicated by red and blue dotted circles in **b**, respectively. Data expressed as the fold increase relative to that in the

uninjured areas are shown as means ± s.e.m. (n = 10 regions examined over 6 independent experiments). Each dot represents an individual sample. n.s., not significant by two-sided t-test. Source data are provided as a Source Data file. Scale bars: 50 μm (**a**, **b**).

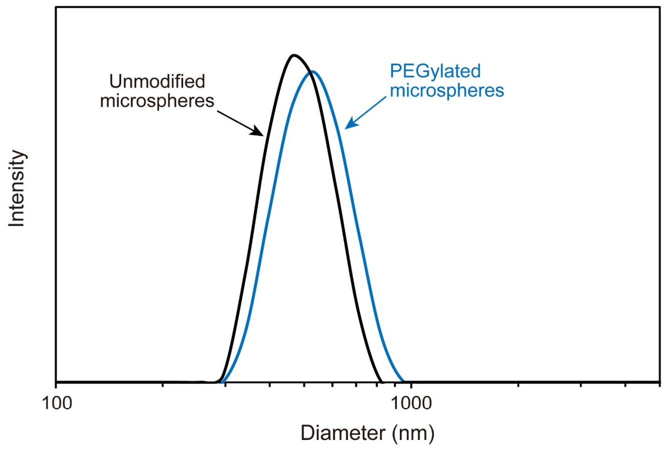

Yuge, Nishiyama et al. Supplementary Figure 3

Supplementary Fig. 3 Size distribution of microspheres. Size distribution of fluorescence microspheres modified with (blue) and without (black) polyethylene glycol was determined by dynamic light scattering (DLS). The data was analyzed by the CONTIN method.

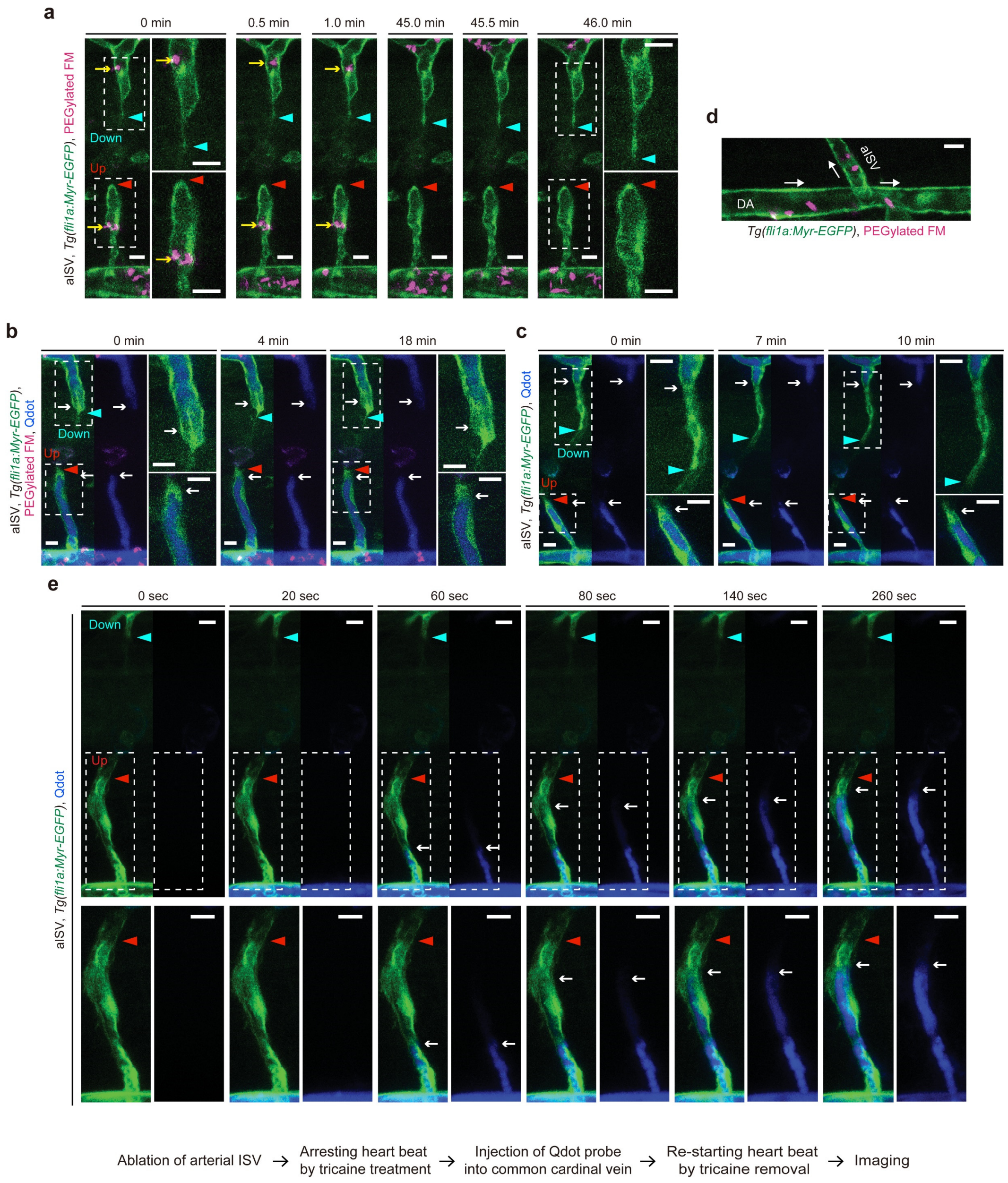

Yuge, Nishiyama et al. Supplementary Figure 4

Supplementary Fig. 4 Hemodynamics in the injured ISVs. a-c Confocal z-projection images of injured aISV in 3 dpf *Tg(fli1a:Myr-EGFP)* larvae intravascularly injected with PEGylated fluorescent microspheres (PEGylated FM) (**a**), with both PEGylated FM and Qdots (Qdot 705) (**b**), and with Qdots (Qdot 705) (**c**) and their subsequent timelapse images at the elapsed time indicated at the top. The imaging was started at 3.0 hpi and 61 min after the injection (**a**), at 2.1 hpi and 15 min after the injection (**b**), and at 2.2 hpi and 84 min after the injection (**c**). Lateral view, anterior to the left. **a** Merged images of EGFP (green) and PEGylated FM (magenta). **b** Left, merged images of EGFP (green), PEGylated FM (magenta) and Qdot (blue); right, merged images of PEGylated

FM and Qdot. **c** Left, merged images of EGFP (green) and Qdot (blue); right, Qdot image. Boxed areas are enlarged on the right. Red and blue arrowheads, leading edges of upstream and downstream injured vessels, respectively (**a**-**c**); white arrows, tip of upstream and downstream injured vessels filled with Qdots (**b**, **c**); yellow arrows; PEGylated FM (**a**). **d** Confocal z-projection image of dorsal aorta and arterial ISV (aISV) in the trunk of 3 dpf *Tg(fli1a:Myr-EGFP)* larva intravascularly injected with PEGylated FM. Lateral view, anterior to the left. Merged image of EGFP (green) and PEGylated FM (magenta). White arrows, direction of blood flow. See also Supplementary Movie 10. **e** Timelapse confocal z-projection images of injured aISV in 3 dpf *Tg(fli1a:Myr-EGFP)* larva intravascularly injected with Qdots (Qdot 655) at the elapsed time indicated at the top. Before imaging, the larva underwent experimental procedure described at the bottom. Initially, a single aISV was injured by laser ablation, and subsequently the heartbeat was arrested by treatment with high concentration of tricaine (0.12-0.13% in E3 imaging medium). After injection of Qdots into the pericardial cavity, the larva was washed with E3 imaging medium to remove tricaine and immediately subjected to timelapse imaging before blood flow started. Lateral view, anterior to the left. Left, merged images of EGFP (green) and Qdot (blue); right, Qdot images. Boxed areas in the upper panel are enlarged on the bottom. Red and blue arrowheads, leading edges of upstream and downstream injured vessels, respectively; white arrows, tip of upstream injured vessels filled with Qdots. See also Supplementary Movies 11. Scale bars: 10 μm.

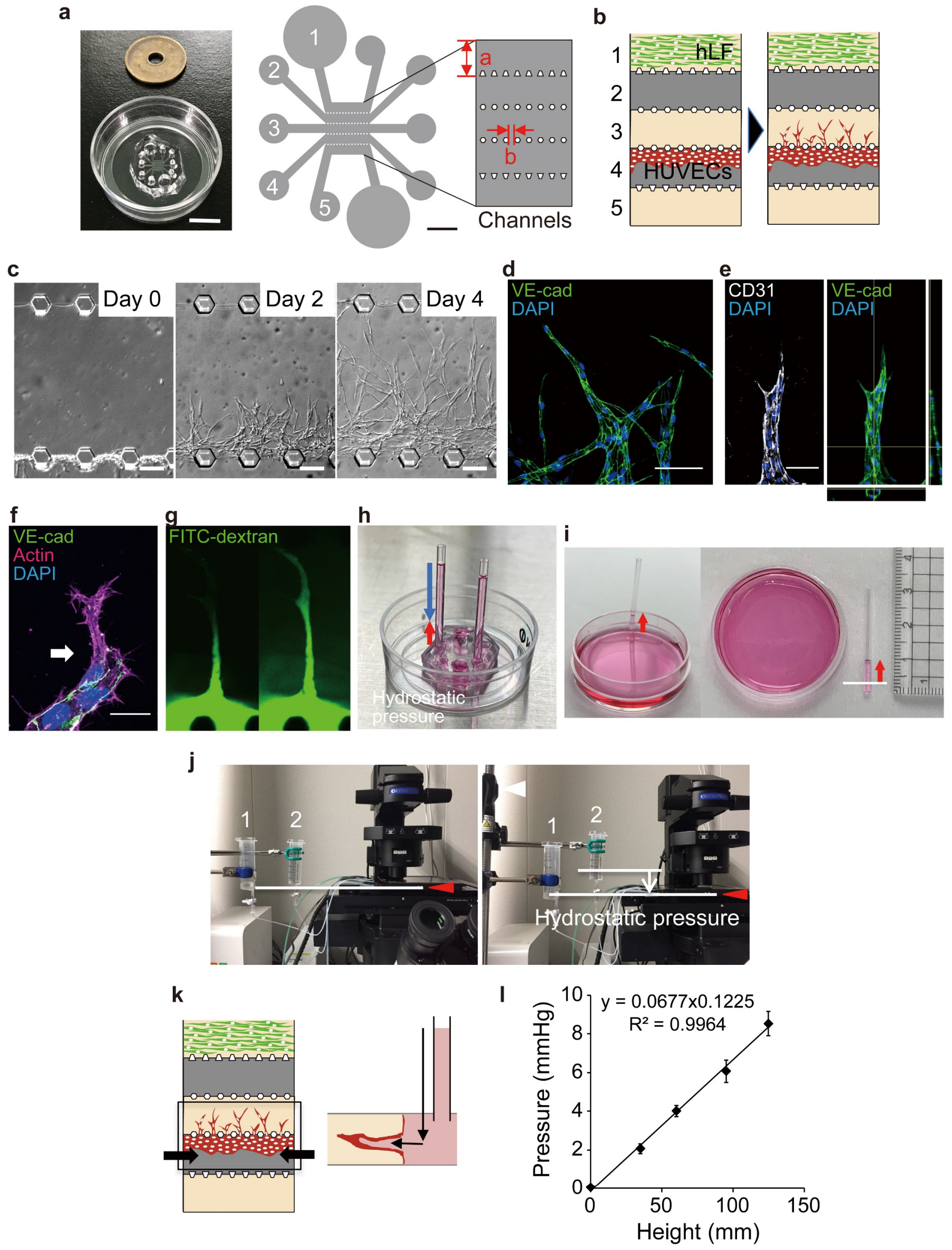

Yuge, Nishiyama et al. Supplementary Figure 5

Supplementary Fig. 5 On-chip angiogenesis assay and vascular intraluminal pressure loading systems. a Microfluidic device mounted on a 35 mm glass bottom culture dish (left). Schematic illustration of top view of the device design (right). Five parallel channels (each 700 µm in width, a) were partitioned by microposts (100 µm window, b). **b** Schematic illustration of on-chip angiogenesis assay. In the on-chip angiogenesis assay, HUVECs seeded on Channel 4 migrate into the fibrin-collagen matrices filling Channel 3 and form angiogenic branches in response to soluble angiogenic factors secreted from human lung fibroblasts (hLF) in Channel 3. **c** Representative DIC images showing on-chip angiogenesis. Angiogenic branches which

formed in Channel 3 before (Day 0) and 2 (Day 2) and 4 (Day 4) days after induction of angiogenesis are shown. **d-f** Confocal z-projection images and the orthogonal views (right in e) of angiogenic sprouts in an on-chip angiogenesis assay. d, the merged image of VE-cadherin (VE-cad, green) and DAPI (blue); e, the merged image of CD31 (white) and DAPI (blue) (left) and that of VE-cadherin (VE-cad, green) and DAPI (blue) (right); f, the merged image of VE-cadherin (VE-cad, green), actin (magenta), and DAPI (blue). Note that an angiogenic sprout has a lumen (**e**). An arrow in **f** indicates a tip EC. **g** Fluorescent images showing visualization of the vascular lumen by introducing FITC-dextran-containing angiogenic medium into channel 4. FITC-dextran diffused from the root of the angiogenic branch (left) and then toward the tip (right) for 1 sec through the lumen. **h-l** Devices for applying hydrostatic pressure to the lumen of elongating vessels. **h**, **i** Fixed type. Hydrostatic pressure (blue arrow in **h**) is applied to the lumens of angiogenic sprouts by placing capillaries filled with media (25 mm) in channel 4. Considering the negative pressure (red arrows in **h** and **i**, approximately 0.6 mmHg) generated by the capillary phenomenon (white line in **i**, water surface in culture dish), approximately 1.2 mmHg hydrostatic pressure is loaded on the lumens of angiogenic branches. **j** Variable type. Hydrostatic pressure was induced by height differences between the water surfaces in syringe 1 and syringe 2. White lines, water surfaces in syringes. **k** Schematic of hydrostatic pressure (arrows) loading system on microfluidic device (left, top view and right, side view). **l** Validation of the variable type of hydrostatic pressure loading system, using a differential pressure gauge. Data are presented as means ± s.e.m. (n = 5 devices examined over 5 independent experiments). Source data are provided as a Source Data file. Scale bars: 10 mm (left in **a**) and 200 µm (right in **a**), 100 µm (**c**, **d**), 20 µm (**e**, **f**).

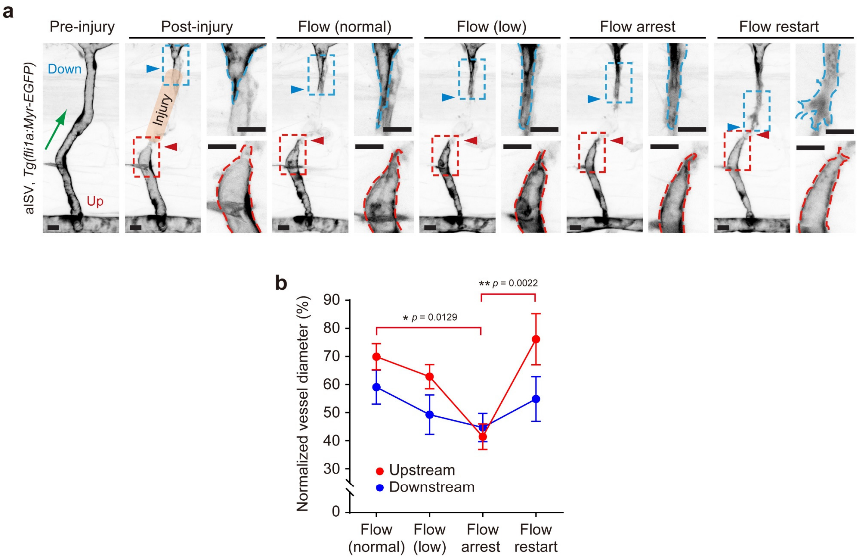

Yuge, Nishiyama et al. Supplementary Figure 6

Supplementary Fig. 6 Effects of changes in blood flow on the morphology of injured aISVs. a Confocal z-projection images of pre- (Pre-injury) and post-injured (Post-injury) aISVs in the *Tg(fli1a:Myr-EGFP)* larval zebrafish at 3 dpf and its subsequent images obtained at the time as follows: at 2.5 hpi [Flow (normal)], when blood flow slowed down [Flow (low)] and was arrested (Flow arrest) by treatment with BDM, when blood flow restarted (Flow restart). Lateral view, anterior to the left. Myr-EGFP images are shown. Orange area, injured region; red and blue arrowheads, leading edges of the upstream and downstream injured aISV, respectively; green arrow, direction of blood flow. The boxed areas are enlarged on the right. Scale bars: 10 µm. **b** Outer diameters of the upstream (red) and downstream (blue) injured aISVs at 10 µm from the leading edge at four time points as indicated in **a**: Flow (normal), Flow (low) (0.6-0.9 h after the beginning of BDM treatment), Flow arrest (2.3-2.5 h after the beginning of BDM treatment), Flow restart (1.9-2.5 h after removing BDM). Outer diameters are shown as a percentage relative to that of the pre-injured aISVs. Data are shown as means ± s.e.m. ($n = 7$ animals). * $p < 0.05$, ** $p < 0.01$ by one-way ANOVA followed by Tukey's test among the changes in the upstream or downstream vessel. Source data are provided as a Source Data file.

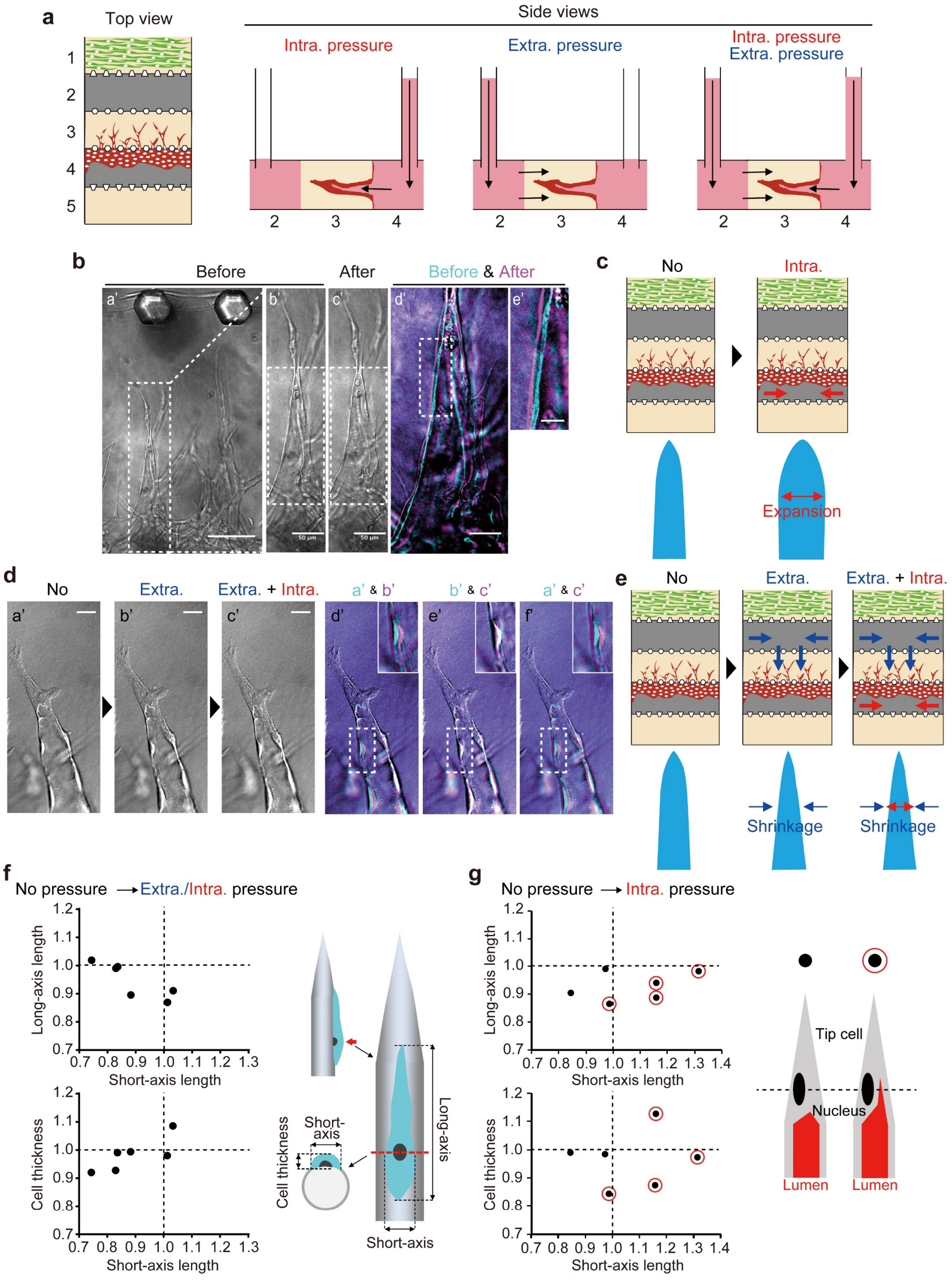

Yuge, Nishiyama et al. Supplementary Figure 7

Supplementary Fig. 7 Acute morphological changes in on-chip angiogenic branches upon loading of intraluminal and extraluminal pressure. a Schematic diagram showing the experimental protocols for applying intraluminal pressure (IP), extraluminal pressure (EP) and both IP and EP to on-chip angiogenic branches. IP, EP and both IP and EP were loaded on angiogenic branches by placing capillaries filled with media in channel 4, channel 2, and both channel 2 and channel 4, respectively. **b**, **c** Acute expansion of angiogenic branches after IP loading (Intra). **b** DIC images showing

on-chip angiogenic branches before and after IP loading. Magnified views of boxed area in image a' before and after IP loading are shown in images b' and c', respectively. Pseudo-colored images of boxed areas in b' (cyan) and c' (magenta) are merged in image d'. Boxed area in d' is enlarged at right (e'). Note that IP loading immediately induced expansion of angiogenic branches. See also Supplementary Movie 13. **c** Schematic explanation for **b**. **d**, **e** Acute morphological changes of angiogenic branches after sequential loading of EP and IP. **d** DIC images showing on-chip angiogenic branch before (a', No) and after (b', Extra) EP load, and after subsequent loading of IP (c', Extra and Intra). Image d', merged image of pseudo-colored images of a' (cyan) and b' (magenta); Image e', merged image of pseudo-colored images of b' (cyan) and c' (magenta); Image f', merged image of pseudo-colored images of a' (cyan) and c' (magenta). Enlarged images of the boxed areas in d', e', and f' are shown in the insets. Note that the angiogenic branch shows abrupt shrinkage after EP loading, which persisted even after subsequent IP loading. See also Supplementary Movies 14 and 15. **e** Schematic explanation for **d**. **f**, **g** Acute morphological changes of stalk (**f**) and tip (**g**) ECs comprising on-chip angiogenic branches after the application of pressure loads were analyzed as described in Fig. 3k-n. **f** Changes in Short- and Long-axis lengths of stalk cells (upper) and those in Short-axis length and Cell thickness of stalk cells (lower) upon sequential loading of EP and IP were measured as shown in the illustration at right, and expressed as a ratio of the values before and after the pressure loads. Each dot represents an individual cell (n = 6 independent experiments). Note that none of the parameters change significantly in response to pressure loads (Long axis: from 183.8 ± 15.8 to 175.1 ±18.2 μm, p = 0.10; Short axis: from 26.3 ± 4.2 to 23.0 ± 3.4 μm, p = 0.06; cell thickness: from 11.7 ± 0.9 to 11.4 ± 0.8 μm, p = 0.45, means ± s.e.m, μm), although short- and long-axis lengths tended to be reduced by pressure loads. **g** Changes in Short- and Long-axis lengths of tip cells (upper) and those in the Short-axis length and the Cell thickness of tip cells (lower) upon IP loading were expressed as a ratio of the values before and after IP loading. Each dot represents an individual cell (n = 6 independent experiments). The dots marked by red circles indicate the tip cells that contribute to lumen formation as shown in the illustration at right. Note that tip cells forming the lumen tend to be stretched by IP loading. Source data are provided as a Source Data file. Scale bars, 100 μm (**b**-a'), 50 μm (**b**-b', **b**-c'), 25 μm (**b**-d'), 10 μm (**b**-e'), 25 μm (**d**).

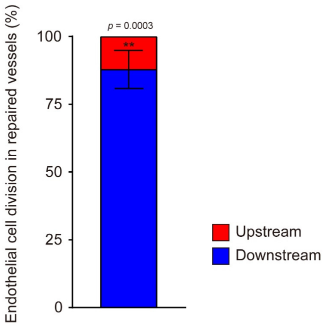

Yuge, Nishiyama et al. Supplementary Figure 8

Supplementary Fig. 8 Proportion of EC division in upstream and downstream injured skin vessels during their repair process. Number of EC division in the upstream (red) and downstream (blue) injured skin vessels during their repair processes are normalized to the elongation length of each vessel and expressed as a percentage of total number of EC division. Data are shown as means ± s.e.m. (n = 4 animals). **p < 0.01 by two-sided t-test. Source data are provided as a Source Data file.

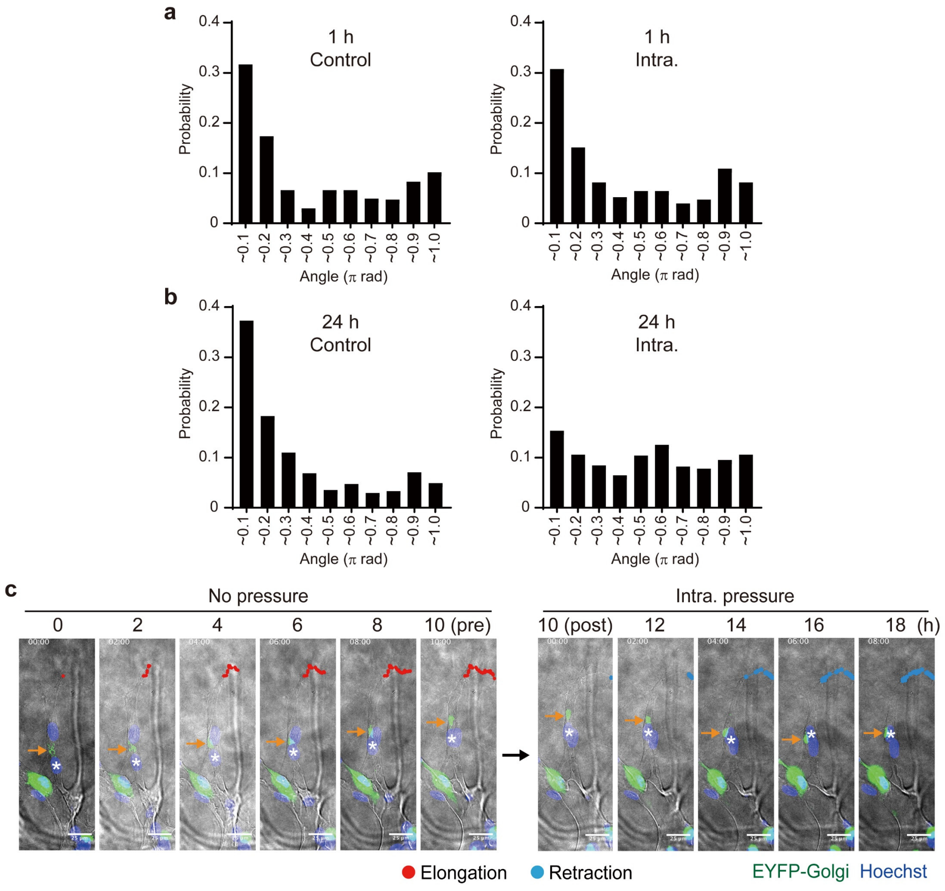

Yuge, Nishiyama et al. Supplementary Figure 9

Supplementary Fig. 9 Impact of IP loading on front-rear polarity of ECs in on-chip angiogenic branches. a, **b** Front-rear polarities of ECs in the on-chip angiogenic branches loaded without (Control) or with IP (Intra.) for 1 h (**a**) and 24 h (**b**) were evaluated by the angle ($0 \leq \theta \leq 1\pi$ rad) between the vector of branch elongation and the vector from the center of the nucleus toward the center of the Golgi apparatus as described in Fig. 4b. Histograms showing the probability distribution of the presence of ECs with the angle indicated at the bottom. Note that the majority of ECs showed positioning of their Golgi apparatus ahead of the nucleus in the direction of branch elongation (between 0 and 0.2π) (Control in **a** and **b**), while the polarized distribution of the Golgi apparatus was randomized upon IP loading for 24 h (Intra. in **b**) but not for 1 h (Intra. in **a**), as evaluated by the Kolmogorov-Smirnov goodness-of-fit test. **a**, Control and Intra., n = 530 and 403 ECs examined over 2 independent experiments; **b**, Control and Intra., n = 509 and 462 ECs examined over 3 independent experiments. **c** Dynamic changes in the position of the Golgi apparatus in the EC of an on-chip angiogenic branch with IP loading. Merged fluorescence and DIC images of the on-chip angiogenic branch before (No pressure) and after (Intraluminal pressure) IP loading at the elapsed time indicated at the top. Green, EYFP-Golgi; blue, Hoechst 33342. Orange arrows and white asterisks show the positions of the Golgi apparatus and the nucleus in an EC,

respectively. Red and blue lines indicate the trajectory of the tip of elongating and retracting angiogenic branches, respectively. Note that in the elongating angiogenic branch, an EC is positioned at the Golgi apparatus ahead of the nucleus toward the direction of branch elongation, while IP loading immediately induced branch retraction and inhibition of polarized positioning of the Golgi apparatus. See also Supplementary Movies 18 and 19. Source data are provided as a Source Data file. Scale bars, 25 μm (**c**).

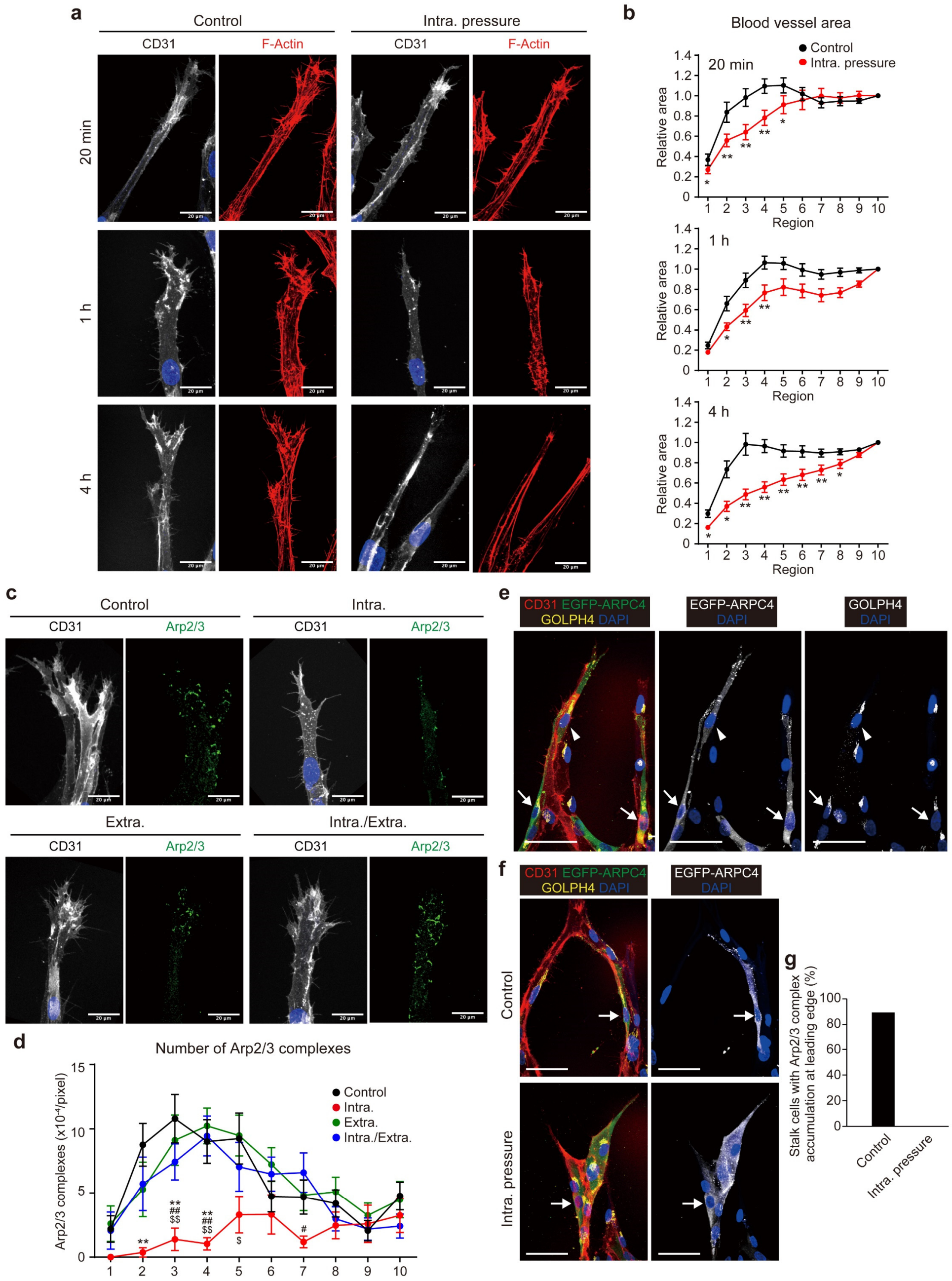

Yuge, Nishiyama et al. Supplementary Figure 10

Supplementary Fig. 10 Impacts of intraluminal and extraluminal pressure loads on leading edge morphology, Arp2/3 complex localization, and F-actin formation in on-chip angiogenic branches. a Confocal z-projection images of angiogenic branches loaded without (Control) and with (Intra. pressure) IP for the time indicated on the left. Left, CD31; right, F-actin. **b** Quantification of leading-edge areas of angiogenic branches as observed in **a** is shown as described in Fig. 7l. Data are means ± s.e.m. (the number of branches examined over 3 independent experiments: 20 min, $n = 27$ and 27; 1 h, $n = 36$ and 25; 4 h, $n = 30$ and 28, for Control and IP, respectively). $^*p < 0.05$. $^{**}p < 0.01$ versus Control by the Mann-Whitney two-sided U test. Note that leading edges

of angiogenic branches gradually became tapered after IP loading. **c** Confocal z-projection images of angiogenic fronts without (Control) and with the indicated pressure load for 1 h. Left, CD31 (grey) and DAPI (blue); right, ARPC2 (Arp2/3) (green). **d** Quantification of the number of Arp2/3 complexes in angiogenic fronts as observed in **c** is shown as described in Fig. 4f. Data are means ± s.e.m. (the number of branches examined over 3 independent experiments: Control, $n = 21$; Intra., $n = 17$; Extra., $n = 18$; Intra./Extra., $n = 23$). **$p < 0.01$ versus Control, #$p < 0.05$, ##$p < 0.01$ versus Extra., \$$p < 0.05$, \$\$$p < 0.01$, versus Intra./Extra. by the Steel-Dwass test. Note that the characteristic localization of Arp2/3 complexes at the leading edge disappeared upon IP loading, but not upon EP loading or both IP and EP loading. **e** Confocal z-projection images of angiogenic branches in which a small population of ECs expresses EGFP-ARPC4. In left image, red, CD31; green, EGFP-ARPC4; yellow, GOLPH4; blue, DAPI. Arrowheads and arrows show tip and stalk cells, respectively. Note that not only tip cells but also stalk cells exhibit localization of EGFP-ARPC4-labeled Arp2/3 complexes at the leading edge. **f** Confocal z-projection images of angiogenic branches without (Control) and with (Intra. pressure) IP loading for 4 h are shown as in **e**. **g** The percentage of stalk cells exhibiting leading edge localization of EGFP-ARPC4-labeled Arp2/3 complexes is the same as that observed in **f** (the number of branches examined over 1 experiment: Control, $n = 9$; Intra. pressure, $n = 7$). Note that IP loading inhibited leading edge localization of Arp2/3 complexes in the stalk cells constituting angiogenic branches. Source data are provided as a Source Data file. Scale bars, 20 µm (**a**, **c**), 50 µm (**e**, **f**).

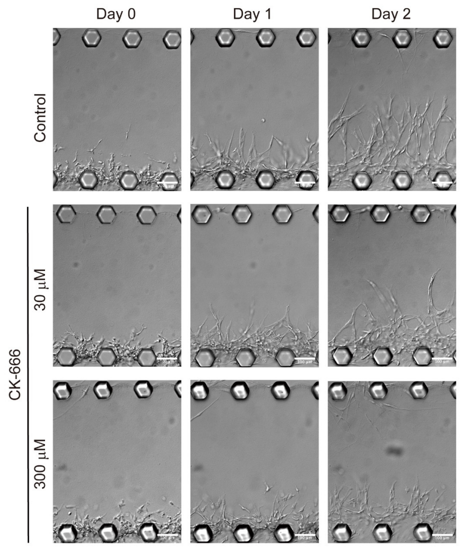

Yuge, Nishiyama et al. Supplementary Figure 11

Supplementary Fig. 11 Effects of CK-666, an inhibitor of the Arp2/3 complex, on branch elongation of on-chip angiogenesis. Representative serial DIC images of angiogenic branch elongation in the absence (Control) or presence (CK-666) of the indicated concentrations of CK-666, an Arp2/3 complex inhibitor, are shown as in Fig. 2e. Quantitative data are shown in Fig. 4g. Note that CK-666 treatment dose-dependently inhibited branch elongation. Scale bars, 100 μm.

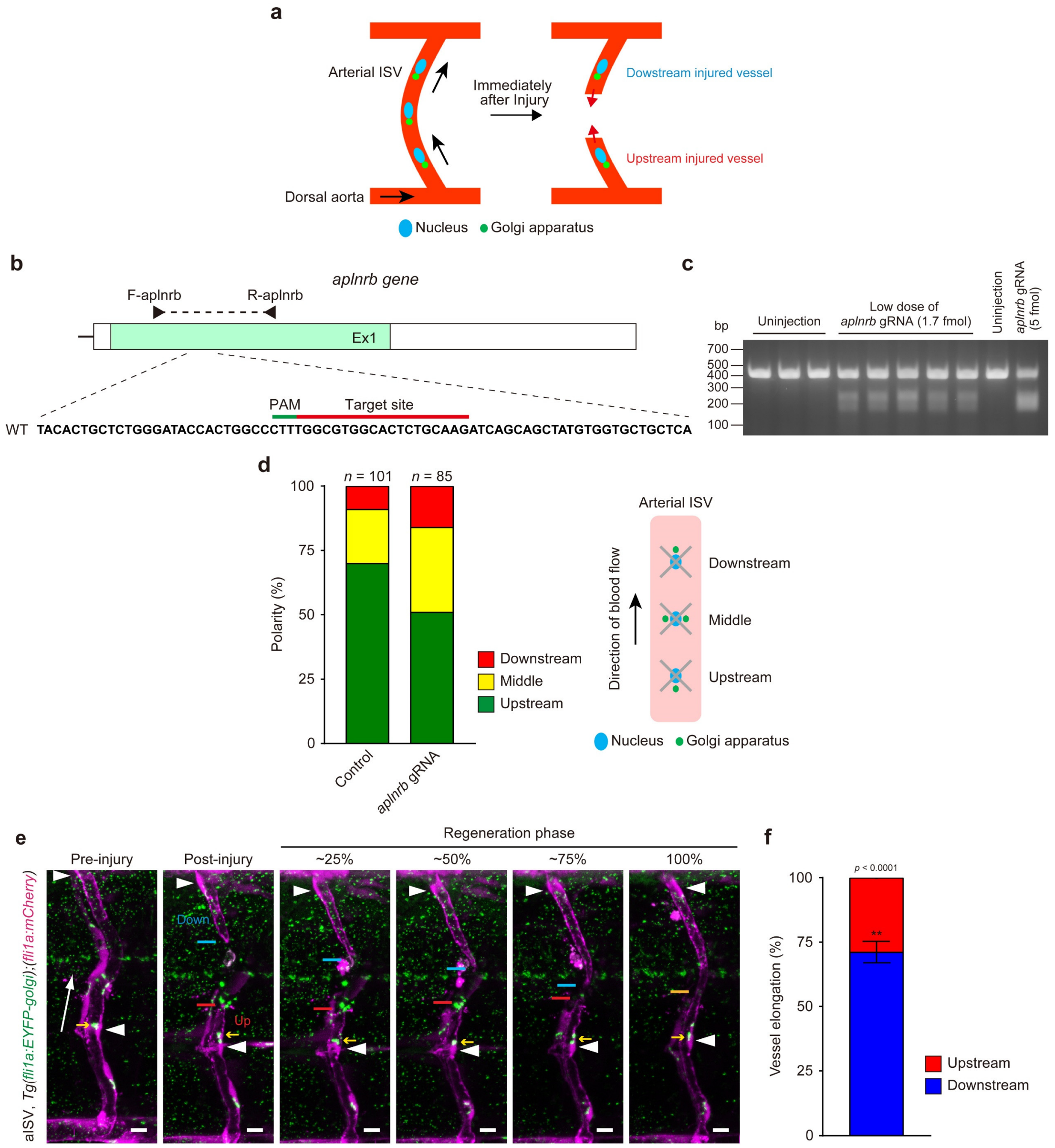

Yuge, Nishiyama et al. Supplementary Figure 12

Supplementary Fig. 12 Elongation of upstream and downstream injured aISVa in zebrafish larvae exhibiting defective EC polarization by blood flow. a Schematic illustration showing front-rear polarity of ECs in aISV before and immediately after injury. Arrows indicate direction of blood flow. **b** Schematic representation of the endogenous *aplnrb* locus and partial sequence of exon 1 of the *aplnrb* gene. Red overline indicates CRISPR guide RNA target site. **c** CRISPR/Cas9-mediated knockout efficiency of *aplnrb* gene in zebrafish. PCR products amplified from genomic DNAs derived from 3 dpf larvae injected without (Uninjected) or with the indicated amounts of *aplnrb* dgRNA:Cas9 RNP complex using the F-aplnrb and R-aplnrb primers indicated by arrowheads in **b** were subjected to T7EI cleavage assay as described in the

Methods. Note that PCR fragments for larvae injected with *aplnrb* dgRNA:Cas9 RNP complex were partially digested by T7 Endonuclease I, indicating that injection of low-dose *aplnrb* dgRNA:Cas9 RNP complex results in partial knockout of the *aplnrb* gene. For an example of presentation of full scan gels, see the Source Data file. **d** Blood flow-mediated establishment of front-rear polarity of ECs in the *Tg(fli1a:h2b-mCherry);(fli1a:EYFP-Golgi)* zebrafish larvae injected without (Uninjected) and with low-dose *aplnrb* dgRNA:Cas9 RNP complex (*aplnrb* gRNA) at 3 dpf. Polarization patterns of ECs were classified into three groups as follows: "Upstream", "Middle" and "Downstream", when the Golgi apparatus was located in the front, middle, and back of the nucleus against the direction of blood flow, respectively, as depicted at the right. Proportion of each polarization pattern is expressed as a percentage of total number of ECs analyzed. **e** Confocal z-projection images of pre- and post-injured aISVs in the *Tg(fli1a:EYFP-Golgi);(fli1a:mCherry)* larva injected with low-dose *aplnrb* dgRNA:Cas9 RNP complex at 3 dpf and its subsequent time-lapse images are shown as in Fig. 5a. Arrowheads, nuclei; yellow arrows, Golgi apparatus in EC located at the tip of upstream injured vessel; white arrow, direction of blood flow; red and blue bars, the leading edge of the upstream and downstream injured aISV, respectively. Note that the EC in the tip of upstream injured aISV positioned its Golgi apparatus ahead of the nucleus toward the elongation direction for vessel repair immediately after injury. Scale bars: 10 μm. See also Supplemental Movie 21. **f** Amounts of elongation of the upstream (red) and downstream (blue) injured aISVs as observed in **e** are expressed as a percentage of the total elongation (measured as length). Only larvae in which ECs in the tip of upstream injured vessels positioned their Golgi apparatus in front or middle of the nucleus toward the vessel elongation direction were analyzed. Note that elongation of injured aISVs was preferentially induced at a side downstream from blood flow even if blood flow-mediated EC polarization was impaired. Data are shown as means ± s.e.m. (n = 10 animals). $^{**}p < 0.01$ by two-sided t-test. Source data are provided as a Source Data file.

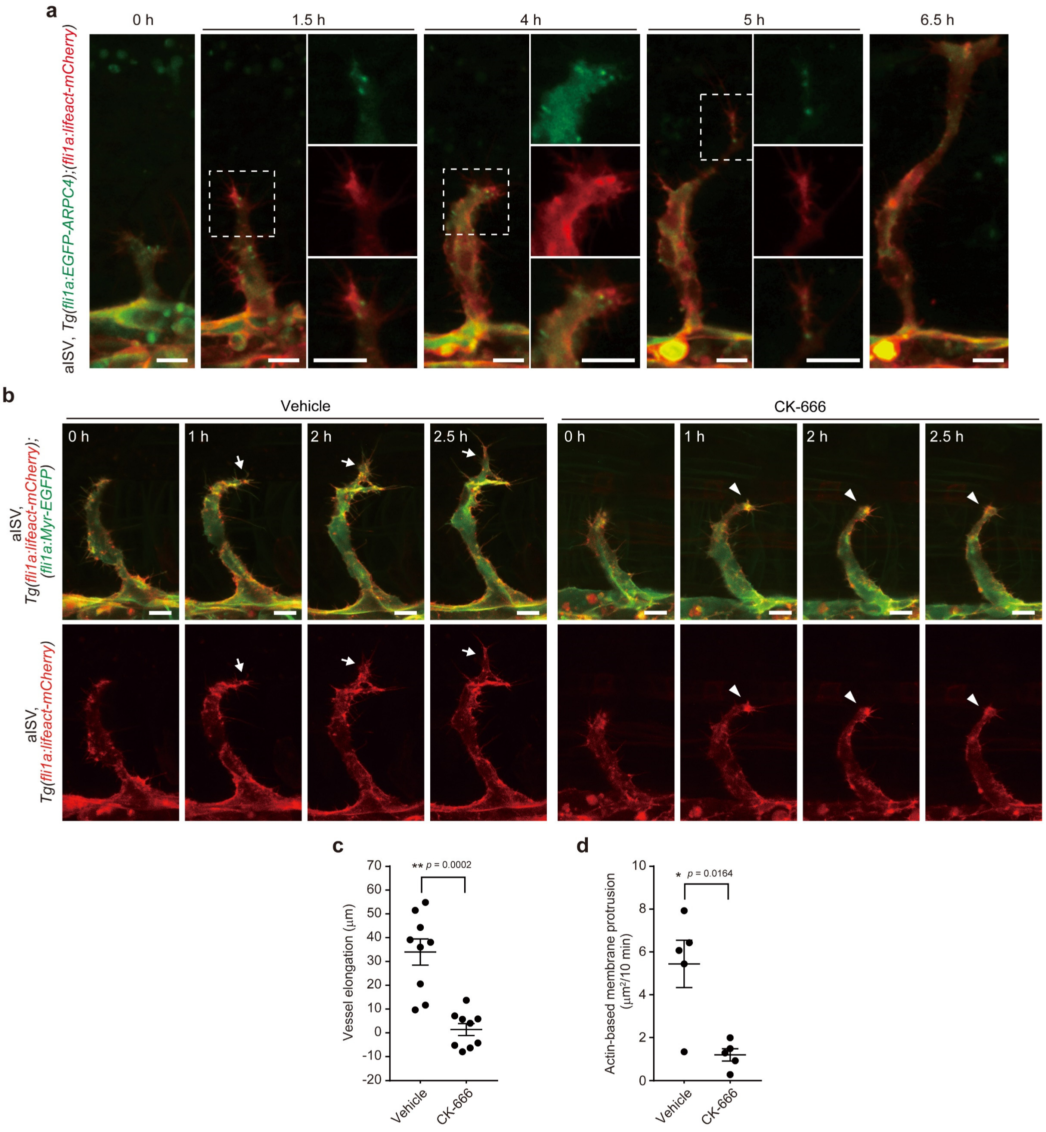

Yuge, Nishiyama et al. Supplementary Figure 13

Supplementary Fig. 13 Inhibitory effect of CK-666, an inhibitor of the Arp2/3 complex, on ISV development in zebrafish embryos. a Confocal z-projection images of ISVs in the *Tg(fli1a:EGFP-ARPC4);(fli1a:lifeact-mCherry)* embryos at 23 hpf and subsequent time-lapse images at the elapsed time indicated at the top. Lateral view, anterior to the left. Merged images of EGFP-ARPC4 (green) and Lifeact-mCherry (red) fluorescence are shown. GFP (top), mCherry (middle), and their merged (bottom) images corresponding to the boxed areas are enlarged on the right. See also Supplementary Movie 23. **b** Time-lapse images of ISV angiogenesis in the presence of vehicle (left) and 200 µM CK-666 (right). Confocal z-projection images of ISVs in the *Tg(fli1a:lifeact-mCherry);(fli1a:Myr-EGFP)* embryos at 24 hpf and subsequent time-lapse images at the elapsed time indicated at the upper left. Treatment with vehicle or CK-666 was started just before imaging. Lateral view, anterior to the left. Upper panels,

the merged images of Lifeact-mCherry (red) and Myr-EGFP (green) fluorescence; lower panels, fluorescence images of Lifeact-mCherry. Arrows indicate formation of actin-based membrane protrusions, whereas arrowheads show the leading edge of angiogenic sprouts that failed to extend membrane protrusions. See also Supplementary Movie 24. **c** Amounts of elongation of ISVs in the presence of vehicle and CK-666 from 24-27 hpf as observed in **b**. Each dot represents an individual ISV. Data are means ± s.e.m. ($n = 9$ animals). **$p < 0.01$ by Welch's two-sided t-test. **d** Quantification of actin-based membrane protrusion formation in the presence of vehicle and CK-666 as observed in **b**. Each dot represents an individual ISV. Values are expressed as an average protruding area per 10 min during 1 h after the beginning of image recording, and shown as means ± s.e.m. ($n = 5$ animals). *$p < 0.05$ by Welch's two-sided t-test. Source data are provided as a Source Data file. Scale bars, 10 µm (**a**, **b**).

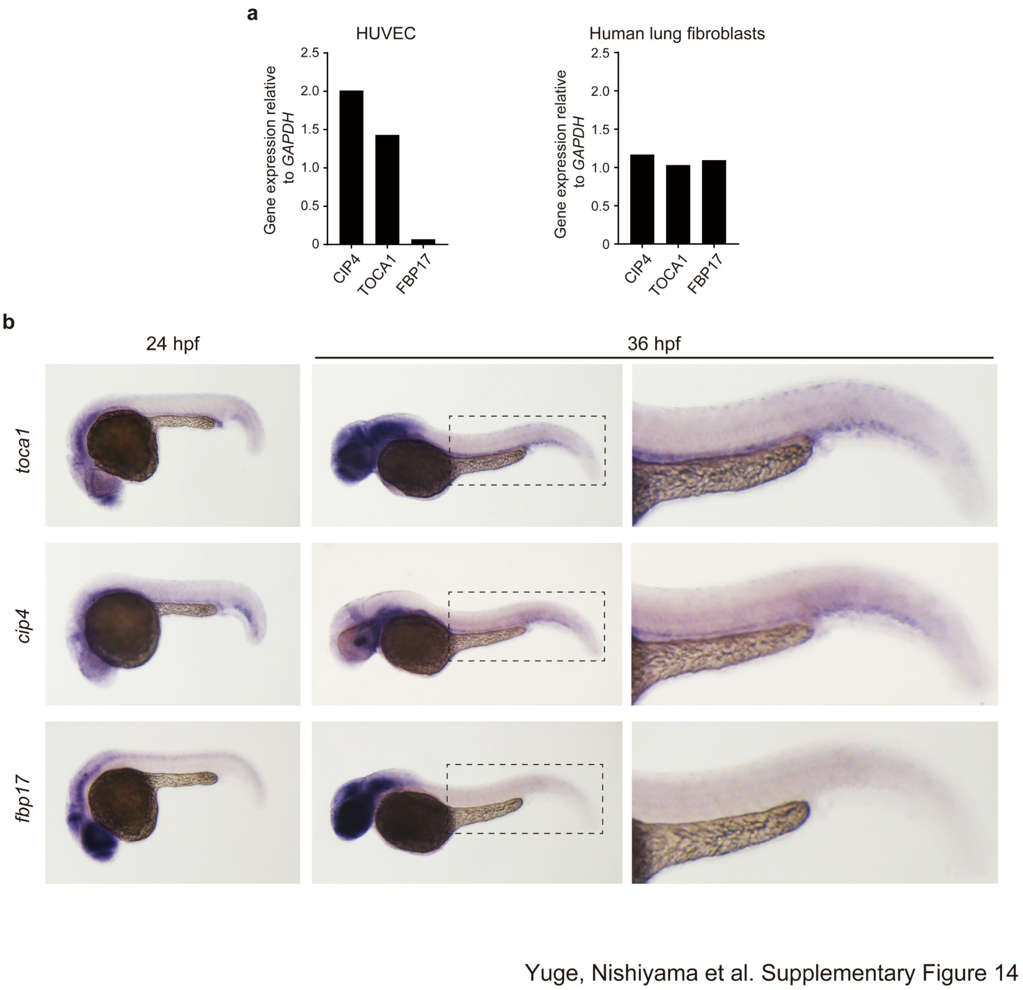

Supplementary Fig. 14 Expression patterns of *toca1*, *cip4*, and *fbp17* in zebrafish embryos. a Expressions of TOCA family genes in HUVECs (left) and human lung fibroblasts (right) analyzed by quantitative PCR relative to that of *GAPDH* (*n* = 1 experiment). **b** Whole-mount *in situ* hybridization of *toca1*, *cip4*, and *fbp17* in zebrafish embryos at 24 and 36 hpf. The boxed areas are enlarged on the right. Note that *toca1* and *cip4*, but not that of *fbp17*, were predominantly expressed in blood vessels. Source data are provided as a Source Data file.

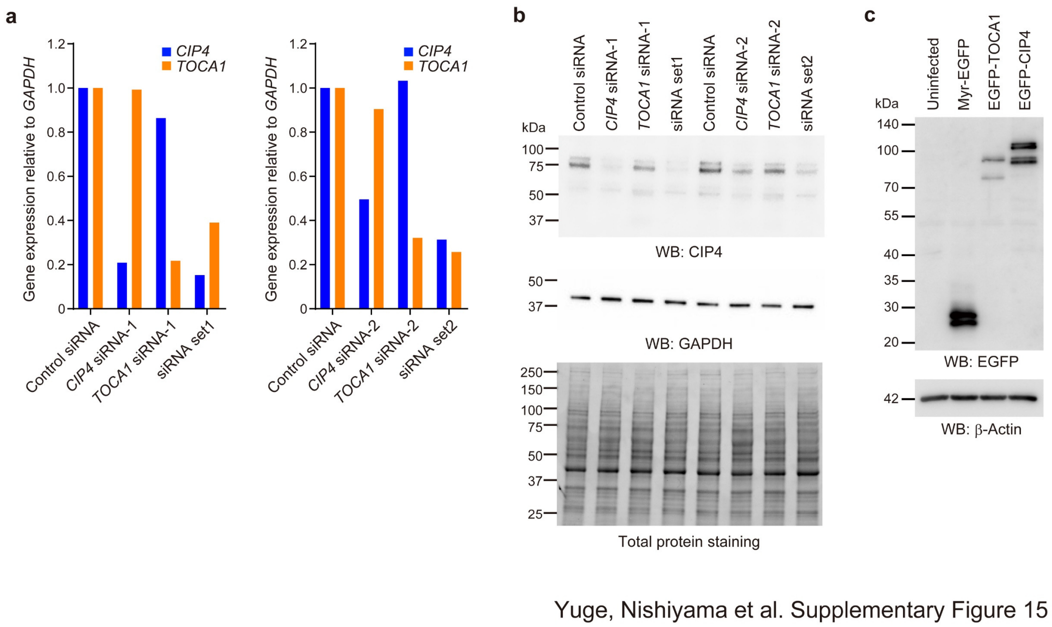

Supplementary Fig. 15 Knockdown of *CIP4* and *TOCA1* by siRNAs and lentivirus-mediated expression of EGFP-TOCA1 and EGFP-CIP4. a Expression levels of *CIP4* and *TOCA1* in HUVECs transfected with siRNAs for *CIP4* and *TOCA1* as indicated at the bottom. Two different sets of siRNA mixtures were used to knockdown both *CIP4* and *TOCA1*; siRNA set1 contains *CIP4* siRNA-1 and *TOCA1* siRNA-1 (left), while siRNA set2 contains *CIP4* siRNA-2 and *TOCA1* siRNA-2 (right). Bar graphs show relative mRNA levels of *CIP4* (blue bars) and *TOCA1* (orange bars) normalized to that of *GAPDH*. Data are expressed relative to that in HUVECs transfected with control siRNA (n = 1 experiment). For the transfection of siRNA targeting either *CIP4* or *TOCA1*, the concentration of total siRNAs was adjusted by including control siRNA. **b** Lysates prepared from HUVECs transfected with the siRNA indicated at the top were subjected to western blot analysis with anti-CIP4 (top) and anti-GAPDH (middle) antibodies. Total proteins visualized by TGX Stain-Free™ FastCast™ Acrylamide Solutions were also shown at the bottom. **c** Lysates prepared from HUVECs infected without (Uninfected) or with lentivirus encoding Myr-EGFP, EGFP-TOCA1, or EGFP-CIP4 were subjected to western blot analysis with anti-EGFP and anti-β-actin antibodies. Source data are provided as a Source Data file. For an example of presentation of full scan blots (**b**, **c**), see the Source Data file.

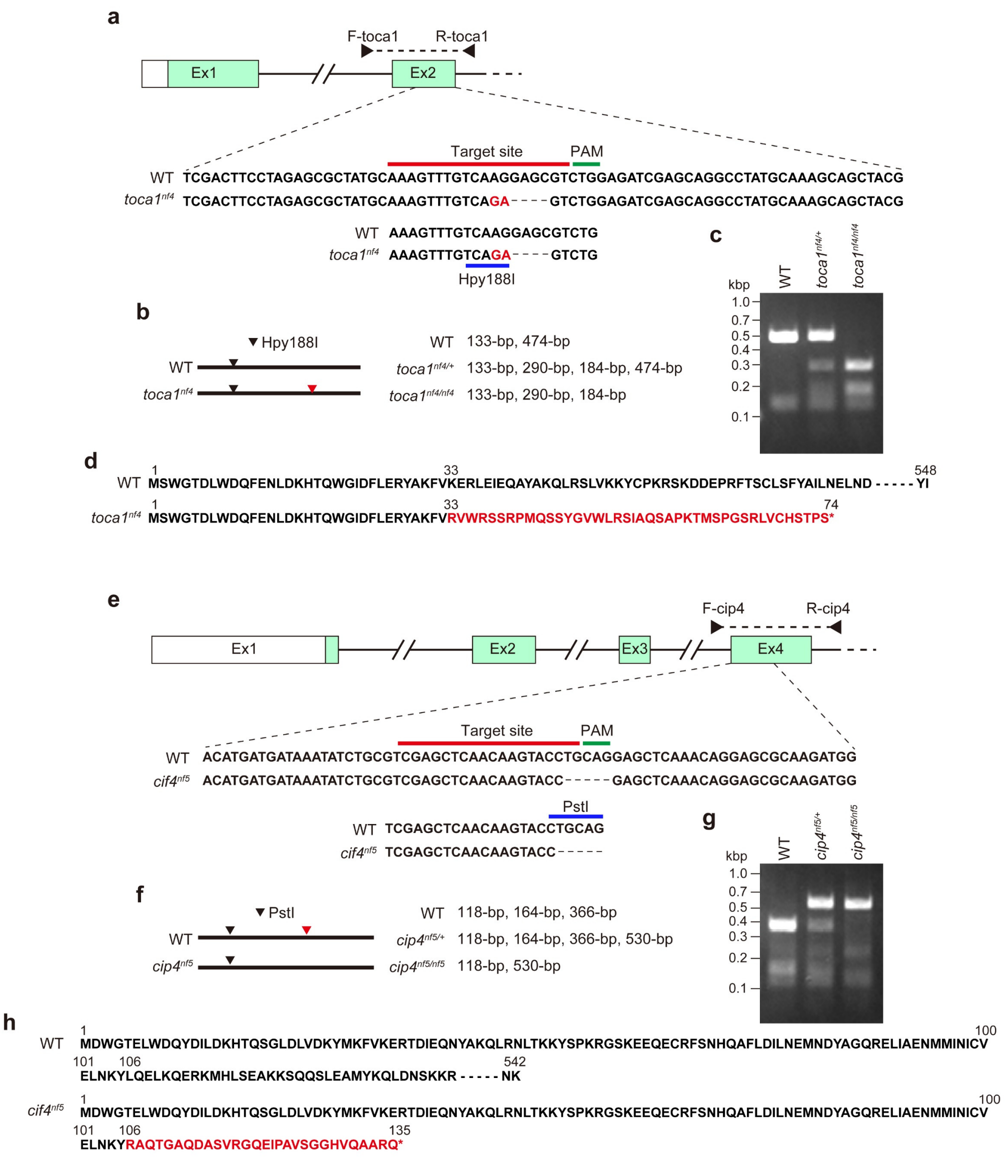

Yuge, Nishiyama et al. Supplementary Figure 16

Supplementary Fig. 16 Generation of *toca1* and *cip4* mutant zebrafish lines. a Schematic representation of the endogenous *toca1* locus and partial sequence of exon 2 of the *toca1* gene in wild type (WT) and *toca1^{nf4}* mutant, showing the CRISPR guide RNA target site in exon 2 (red overline). The *toca1^{nf4}* mutant carries a 5-bp deletion and a 2-bp insertion, creating an additional Hpy188I restriction site (blue underline). **b**, **c** Genotyping of *toca1^{nf4}* mutant. **b** PCR products amplified from genomic DNAs derived from WT and *toca1^{nf4}* mutant (*toca1^{nf4}*) using the F-toca1 and R-toca1 primers indicated by arrowheads in **a**. Black and red downward arrowheads indicate the Hpy188I restriction sites. **c** Genotypes of WT, *toca1$^{nf4/+}$*, and *toca1$^{nf4/nf4}$* as confirmed by digestion of the PCR fragments with Hpy188I, yielding 133-bp and 474-bp fragments for WT, 133-bp, 290-bp, 184-bp, and 474-bp fragments for *toca1$^{nf4/+}$*, and 133-bp, 290-

bp, and 184-bp fragments for *toca1*$^{nf4/nf4}$. **d** Amino acid sequences encoded by WT and the mutated *toca1*^{nf4} genes. Mutated allele in *toca1*^{nf4} encodes 41 mutated amino acids from Lys33, followed by premature stop codons. **e** Schematic representation of the endogenous *cip4* locus and partial sequence of exon 4 of the *cip4* gene in WT and *cip4*^{nf5} mutant, showing the CRISPR guide RNA target site in exon 4 (red overline). The *cip4*^{nf5} mutant carries a 5-bp deletion, removing the PstI restriction site (blue overline). **f**, **g** Genotyping of *cip4*^{nf5} mutant. **f** PCR products amplified from genomic DNAs derived from WT and *cip4*^{nf5} mutant (*cip4*^{nf5}) using the F-cip4 and R-cip4 primers indicated by arrowheads in **e**. Black and red downward arrowheads indicate the PstI restriction sites. **g** Genotypes of WT, *cip4*$^{nf5/+}$, and *cip4*$^{nf5/nf5}$ as confirmed by digestion of the PCR fragments with PstI, yielding 118-bp, 164-bp, and 366-bp fragments for WT, 118-bp, 164-bp, 366-bp, and 530-bp fragments for *cip4*$^{nf5/+}$, and 118-bp and 530-bp fragments for *cip4*$^{nf5/nf5}$. **h** Amino acid sequences encoded by WT and the mutated *cip4*^{nf5} genes. Mutated allele in *cip4*^{nf5} encodes 29 mutated amino acids from Arg106, followed by premature stop codons. For an example of presentation of full scan gels (**c**, **g**), see the Source Data file.

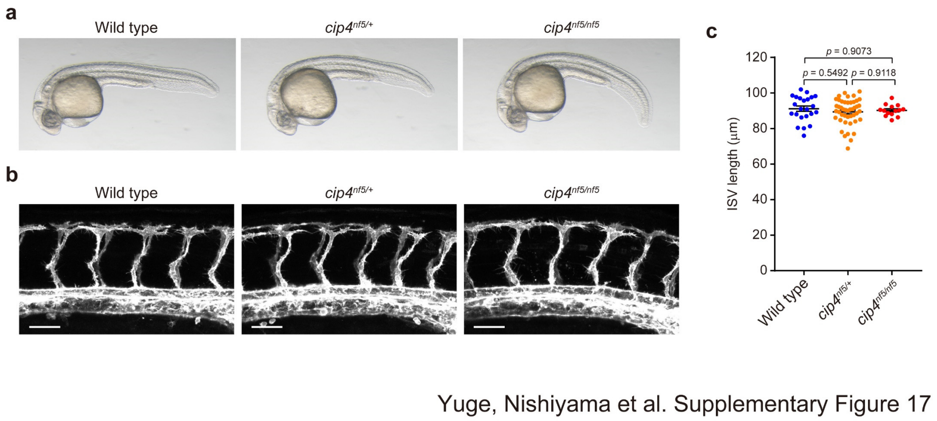

Supplementary Fig. 17 ISV development in *cip4* mutant embryos. a Representative bright field images of WT, *cip4*$^{nf5/+}$, *and cip4*$^{nf5/nf5}$ embryos at 28 hpf. **b** Confocal z-projection images of the trunk vasculature in WT, *cip4*$^{nf5/+}$, *and cip4*$^{nf5/nf5}$ embryos at 28 hpf with the *Tg(fli1a:lifeact-mCherry)* background. Lateral views with anterior to the left. **c** Quantification of ISV length in the WT, *cip4*$^{nf5/+}$, *and cip4*$^{nf5/nf5}$ embryos as observed in **b**. Each dot represents an individual embryo. Data are means ± s.e.m. (WT, n = 23 animals; *cip4*$^{nf5/+}$, n = 46 animals; *cip4*$^{nf5/nf5}$, n = 14 animals). Statistical testing by one-way ANOVA followed by Tukey's test. Note that deletion of *cip4* did not affect ISV development, although it caused mild ventral curvature phenotypes. Source data are provided as a Source Data file. Scale bars, 50 μm (**b**).

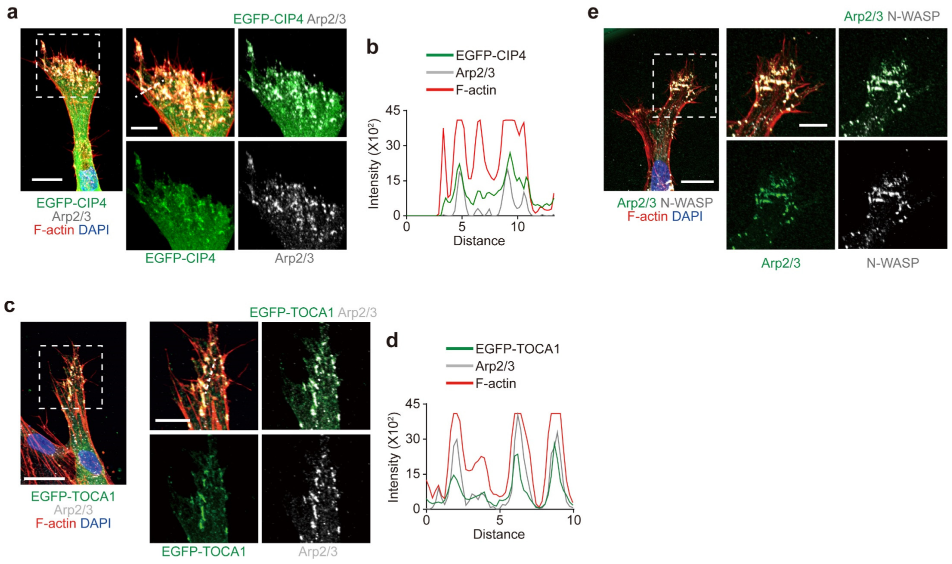

Yuge, Nishiyama et al. Supplementary Figure 18

Supplementary Fig. 18 Localization of EGFP-CIP4, EGFP-TOCA1, Arp2/3 complexes, N-WASP, and F-actin at the leading edge of an on-chip angiogenic branch. **a**, **b** Colocalization of EGFP-CIP4, Arp2/3 complexes, and F-actin at the leading edge of an on-chip angiogenic branch. **a** Confocal z-projection image of an angiogenic branch (left). Green, EGFP-CIP4; gray, ARPC2 (Arp2/3); red, F-actin; blue, DAPI. The boxed area is enlarged on the right. **b** Line scan profiles of fluorescence intensity of EGFP-CIP4 (green), Arp2/3 complex (grey), and F-actin (red) along the dotted line indicated in the merged image in **a**. **c**, **d** Colocalization of EGFP-TOCA1 (green), Arp2/3 complex (grey), and F-actin (red) at the leading edge of an angiogenic branch is shown as in **a** and **b**. **e** Colocalization of ARPC2 (green), N-WASP (grey), and F-actin (red) at the leading edge of an angiogenic branch is shown as in **a**. Scale bars: 20 µm (**a**, **b**, **e**), 10 µm (enlarged images in **a**, **b**, **e**).

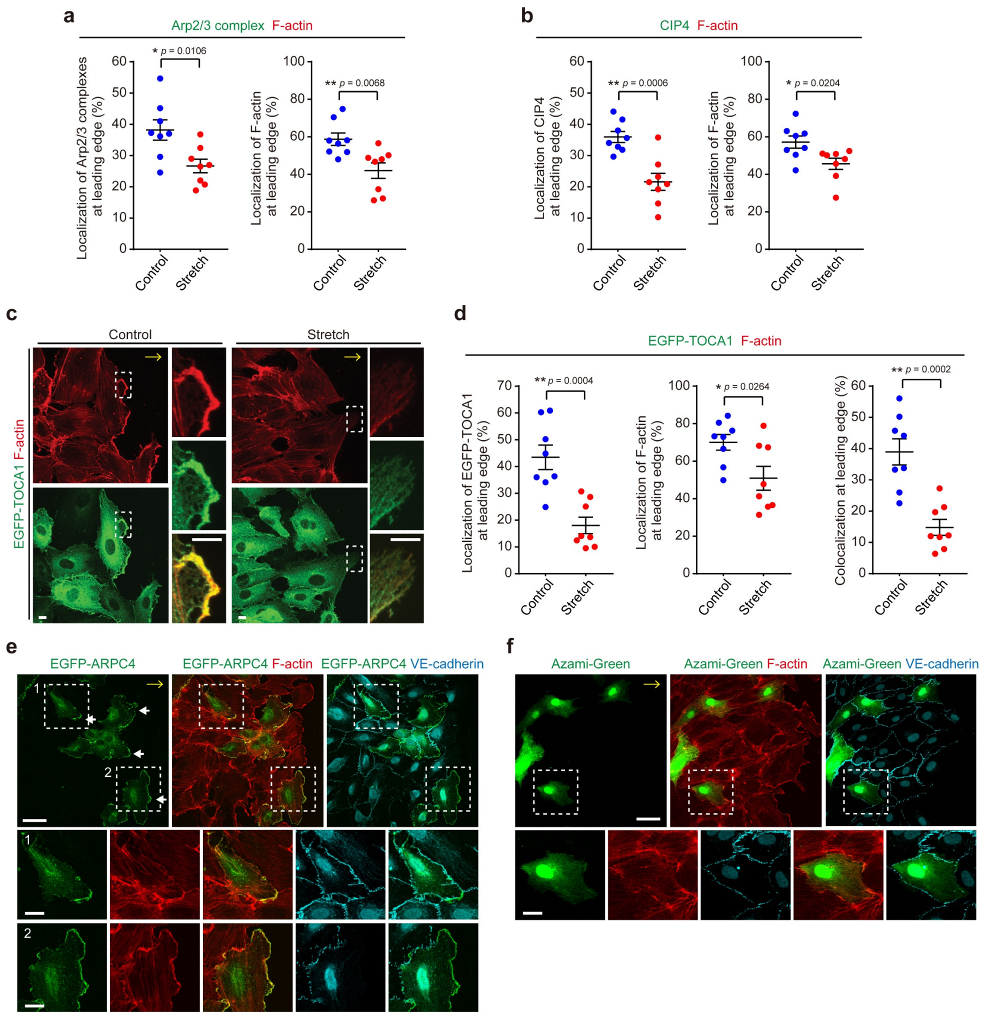

Yuge, Nishiyama et al. Supplementary Figure 19

Supplementary Fig. 19 Effects of EC stretching on localization of Arp2/3 complexes, CIP4, and EGFP-TOCA1 at leading edge of directionally migrating ECs. a, **b** Effect of biaxial stretching on localization of Arp2/3 complexes and CIP4 and amount of F-actin at leading edges of HUVECs directionally migrating on the stretching chambers. Quantification of Arp2/3 complexes (left in **a**), CIP4 (left in **b**), and F-actin (right in **a** and **b**) localized at leading edges of HUVECs as observed in Fig. 8a and c, respectively. Each dot represents an individual confocal image (blue, Control; red Stretch). Data are means ± s.e.m (n = 8 each regions examined over 2 independent experiments for each). *$p < 0.05$, **$p < 0.01$ by two-sided t-test. **c** Effect of biaxial stretching on localization of EGFP-TOCA1 and F-actin at leading edges of HUVECs directionally migrating on the stretching chambers. Confocal z-projection images of HUVECs exposed to continuous biaxial stretch for 3 min after being stretched to 10% over 8 min (Stretch) or kept under static condition (Control). Left upper, F-actin (red); left lower, EGFP-TOCA1. F-actin (upper), EGFP-TOCA1 (middle), and the merged (lower) images of the boxed areas are enlarged on the right. Yellow arrows, direction of

cell migration. **d** Quantification of EGFP-TOCA1 (left) and F-actin (middle) localized at leading edges of HUVECs and that of EGFP-TOCA1 colocalized with F-actin (right) at leading edges of HUVECs as observed in **c**. Each dot represents an individual confocal image (blue, Control; red Stretch). Data are means ± s.e.m (n = 8 each regions examined over 2 independent experiments for each). *$p < 0.05$, **$p < 0.01$ by two-sided t-test. **e**, **f** Confocal z-projection images of directionally migrating HUVECs that mosaically expressed EGFP-ARPC4 (**e**) or Azami-Green (**f**) on the glass-base dishes. Left, EGFP-ARPC4 (green) or Azami-Green (green) image; middle, the merged image of either EGFP-ARPC4 or Azami-Green and F-actin (red); the merged image of either EGFP-ARPC4 or Azami-Green and VE-cadherin (cyan). **e** Representative follower (boxed area 1) and leader (boxed area 2) cells expressing EGFP-ARPC4 are enlarged at the bottom. **f** Representative follower cell expressing Azami-Green indicated by dotted box are enlarged at the bottom. White arrows, leading edge localization of EGFP-ARPC4; yellow arrows, direction of cell migration. Source data are provided as a Source Data file. Scale bars 10 μm (**c**), 50 μm (**e**, **f**), 20 μm (enlarged images in **e**, **f**).

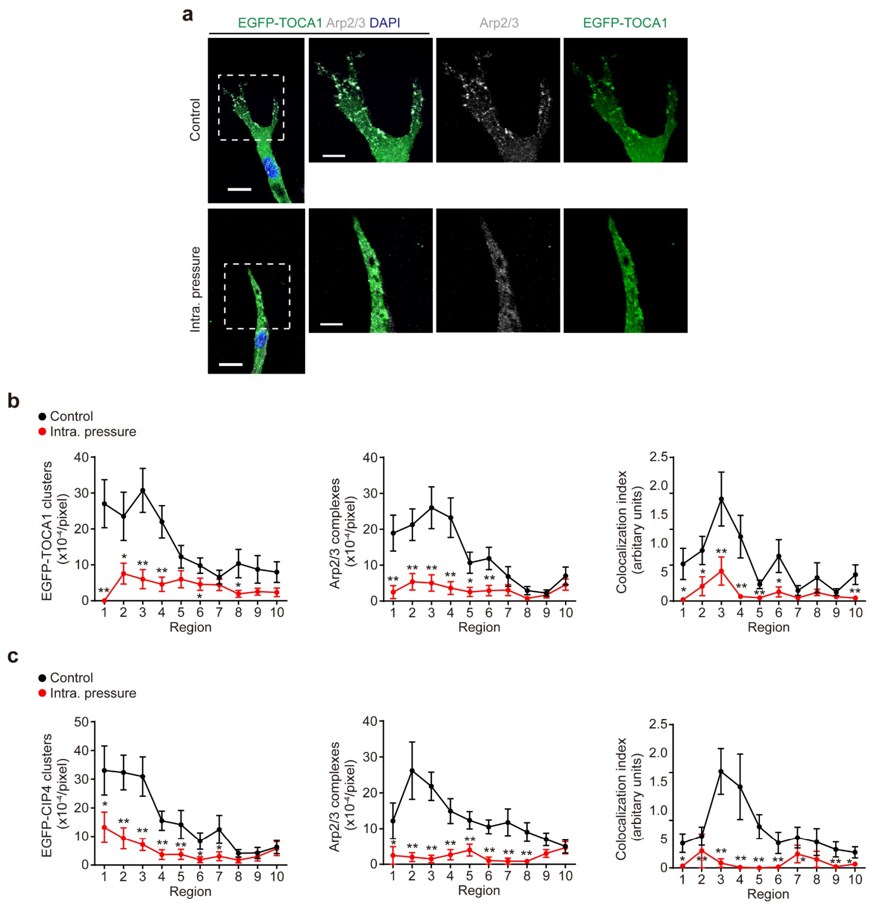

Yuge, Nishiyama et al. Supplementary Figure 20

Supplementary Fig. 20 Effect of IP loading on localization of EGFP-TOCA1, EGFP-CIP4, and Arp2/3 complexes at leading edge of on-chip angiogenic branch. **a** Confocal z-projection images of angiogenic sprouts loaded without (upper, Control) and with IP (lower, Intra. pressure) for 20 min. The boxed areas are enlarged on the right. Green, EGFP-TOCA1; grey, ARPC2 (Arp2/3); blue, DAPI. Scale bars: 25 μm. **b** Quantification of number of EGFP-TOCA1 clusters (left), that of Arp2/3 complexes (middle), and degree of their colocalization (right) in the individual regions of angiogenic branches as observed in **a** are shown as in Fig. 8f. Data are means ± s.e.m (Control and Intra. pressure, n = 22 and 22 branches examined over 3 independent experiments). $*p < 0.05$, $**p < 0.01$ versus Control by the Mann-Whitney two-sided U test. **c** Quantification of number of EGFP-CIP4 clusters (left), that of Arp2/3 complexes (middle), and degree of their colocalization (right) in the individual regions of angiogenic branches are shown as in Fig. 8f. Data are means ± s.e.m (Control and Intra. pressure, n = 18 and 20 branches examined over 3 independent experiments). $*p < 0.05$, $**p < 0.01$ versus Control by the Mann-Whitney two-sided U test. For detailed statistics in **b** and **c**, see Supplementary Table 4. Source data are provided as a Source Data file.

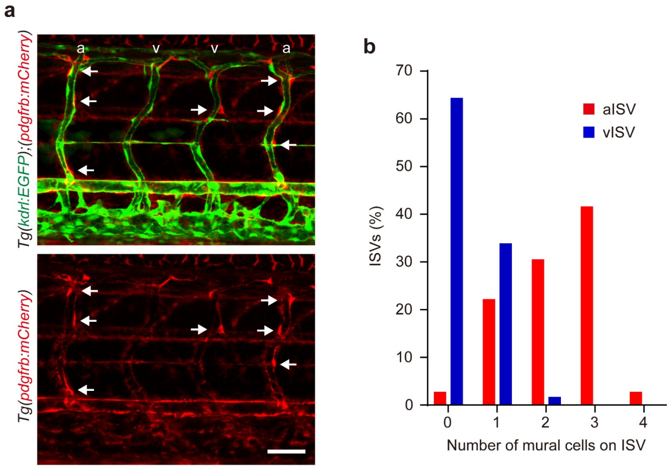

Yuge, Nishiyama et al. Supplementary Figure 21

Supplementary Fig. 21 Mural cell coverage of arterial and venous ISVs in zebrafish larvae. a Confocal z-projection images of ISVs in the trunk of *Tg(kdrl:EGFP);(pdgfrb:mCherry)* larva at 3 dpf. Lateral view, anterior to the left. Upper, the merged image of kdrl:EGFP (green) and pdgfrb:mCherry (red); lower, pdgfrb mCherry image. Arrows, mural cells; a, aISV; v, venous ISV (vISV). Scale bar, 50 µm. **b** Quantification of number of pdgfrb:mCherry-labeled mural cells covering aISVs (red) and vISVs (blue). Proportion of ISVs covered by the indicated number of mural cells is expressed as a percentage of total number of ISVs (aISVs and vISVs, *n* = 36 and 59 vessels examined over 13 animals, respectively). Note that most of aISVs were wrapped by mural cells, while more than 60% of vISVs lacked the coverage of mural cells. Source data are provided as a Source Data file.

Supplemental Tables

Supplementary Table 1. Average diameter and zeta potential of microspheres.

	Diameter (nm)[a]	PDI[b]	Zeta potential (mV)
Unmodified	481 ± 6	0.034 ± 0.04	-50.62 ± 0.79
PEGylated	520 ± 3	0.015 ± 0.01	-18.23 ± 1.47

[a] Determined by DLS. The data was analyzed by the cumulant method.

[b] Polydispersity index. Determined by DLS. The data was analyzed by the cumulant method.

Data are shown as means ± s.e.m. (n = 3 independent experiments).

Supplementary Table 2. Primer sequences for cDNA cloning, genotyping and qPCR.

**Zebrafish cDNA cloning**		
	Forward primer	Reverse primer
toca1 (for full coding sequence)	ATGAGCTGGGGAACGGATCTTTGG	TCATATGTAGGTGACCGCACCTTTGC
toca1 (for probe)	ATGAGCTGGGGAACGGATCTTTGG	GGTCCCAATGGTGCTGTCTGACCC
cip4 (for probe)	ATGGACTGGGGAACTGAGCTTTGG	TAGACTGCTGTCTGATGAGGCCCG
fbp17 (for probe)	ATGCATTCAAACAGAGGATTATCG	TCCACGTCACCCGGCGGCTCGAAG
**Human cDNA cloning**		
	Forward primer	Reverse primer
ARPC4	ATGACTGCCACTCTCCGCCC	TTAAAAATTCTTAAGGAACTCTTC
CIP4	ATGGATTGGGGCACTGAGCTGTG	TTACACAGGTCTCAGCCGAAGCC
**qPCR**		
CIP4	GAAAGAACGCACCGAAGTGGA	TGGAGAATCTGTACGAAGGACTG
TOCA1	GGATCAGTTCGACAGCTTAGAC	AGGCTACACACGAGGTAAACC
FBP17	GCATGAAGTTATCTCCGAGAACA	CGGCCATCGTGAAAGTTTGAT
GAPDH	GGAGCGAGATCCCTCCAAAAT	GGCTGTTGTCATACTTCTCATGG
**Genotyping**		
toca1^{nf4} mutant	ATAAACGTTGTTGGGCAGGA (F-toca1)	TCTGGATCCGTGCATCAGTA (R-toca1)
cip4^{nf5} mutant	TATTTTGGCTGACGCATTCA (F-cip4)	ATGAGCAACGGAAATAGCAA (R-cip4)

Supplementary Table 3. siRNA sequences.

siRNA	Target gene	Sequence
CIP4 siRNA-1	human *CIP4*	5’-CCAAGAACGACUCCCACGUCCUUAU-3’
CIP4 siRNA-2	human *CIP4*	5’-AAGACAUACACGGACACUGAGGUUC-3’
TOCA1 siRNA-1	human *TOCA1*	5’-CAAAGGUGACGGAUGGACAAGAGCU-3’
TOCA1 siRNA-2	human *TOCA1*	5’-GCAGUGACAUAAAUCAUCUUGUAAC-3’

Supplementary Table 4. Detailed statistics.

Figure	Statistics	Comparison	Significance	Adjusted *p* value
Fig. 4e	Two-sided Mann-Whitney U test	20 min		
		Region 1, Cont. vs. Intra. pressure	n.s.	0.8124
		Region 2, Cont. vs. Intra. pressure	n.s.	0.4732
		Region 3, Cont. vs. Intra. pressure	n.s.	0.0979
		Region 4, Cont. vs. Intra. pressure	n.s.	0.0997
		Region 5, Cont. vs. Intra. pressure	n.s.	0.254
		Region 6, Cont. vs. Intra. pressure	n.s.	0.4625
		Region 7, Cont. vs. Intra pressure	n.s.	0.169
		Region 8, Cont. vs. Intra. pressure	*	0.0234
		Region 9, Cont. vs. Intra. pressure	n.s.	0.2132
		Region 10, Cont. vs. Intra. pressure	n.s.	0.8402
		1 h		
		Region 1, Cont. vs. Intra. pressure	n.s.	0.643
		Region 2, Cont. vs. Intra. pressure	n.s.	0.5456
		Region 3, Cont. vs. Intra. pressure	*	0.0357
		Region 4, Cont. vs. Intra. pressure	**	0.0012
		Region 5, Cont. vs. Intra. pressure	**	0.0088
		Region 6, Cont. vs. Intra. pressure	n.s.	0.1384
		Region 7, Cont. vs. Intra. pressure	*	0.037
		Region 8, Cont. vs. Intra. pressure	n.s.	0.7793
		Region 9, Cont. vs. Intra. pressure	n.s.	0.8471
		Region 10, Cont. vs. Intra. pressure	n.s.	0.6809
		4 h		
		Region 1, Cont. vs. Intra. pressure	**	0.0007
		Region 2, Cont. vs. Intra. pressure	**	0.0005
		Region 3, Cont. vs. Intra. pressure	**	0.0004
		Region 4, Cont. vs. Intra. pressure	**	0.0001
		Region 5, Cont. vs. Intra. pressure	**	0.0048
		Region 6, Cont. vs. Intra. pressure	**	0.0037
		Region 7, Cont. vs. Intra. pressure	n.s.	0.1995
		Region 8, Cont. vs. Intra. pressure	n.s.	0.2106
		Region 9, Cont. vs. Intra. pressure	**	0.0054
		Region 10, Cont. vs. Intra. pressure	n.s.	0.0578
	Descriptive	Control 20 min, 1 h, 4 h, n = 27, 36, 30 branches (3 independent experiments)<br>Intra. pressure 20 min, 1 h, 4 h, n = 27, 25, 28 branches (3 independent experiments)		
Fig. 4f	Two-sided Mann-Whitney U test	20 min		
		Region 1, Cont. vs. Intra. pressure	**	0.0014
		Region 2, Cont. vs. Intra. pressure	**	0.001
		Region 3, Cont. vs. Intra. pressure	**	<0.0001
		Region 4, Cont. vs. Intra. pressure	**	<0.0001
		Region 5, Cont. vs. Intra. pressure	**	0.0002
		Region 6, Cont. vs. Intra. pressure	n.s.	0.0527
		Region 7, Cont. vs. Intra. pressure	*	0.0415

		Region 8, Cont. vs. Intra. pressure	n.s.	0.5737
		Region 9, Cont. vs. Intra. pressure	n.s.	0.3313
		Region 10, Cont. vs. Intra. pressure	n.s.	0.0809
		1 h		
		Region 1, Cont. vs. Intra. pressure	n.s.	0.2717
		Region 2, Cont. vs. Intra. pressure	n.s.	0.1147
		Region 3, Cont. vs. Intra. pressure	**	0.0057
		Region 4, Cont. vs. Intra. pressure	**	0.0009
		Region 5, Cont. vs. Intra. pressure	**	0.0097
		Region 6, Cont. vs. Intra. pressure	n.s.	0.5389
		Region 7, Cont. vs. Intra. pressure	**	0.0030
		Region 8, Cont. vs. Intra. pressure	n.s.	0.1432
		Region 9, Cont. vs. Intra. pressure	n.s.	0.2028
		Region 10, Cont. vs. Intra. pressure	n.s.	0.6691
		4 h		
		Region 1, Cont. vs. Intra. pressure	n.s.	0.5624
		Region 2, Cont. vs. Intra. pressure	*	0.0117
		Region 3, Cont. vs. Intra. pressure	**	0.0042
		Region 4, Cont. vs. Intra. pressure	**	0.0027
		Region 5, Cont. vs. Intra. pressure	n.s.	0.4489
		Region 6, Cont. vs. Intra. pressure	*	0.0226
		Region 7, Cont. vs. Intra. pressure	n.s.	0.6594
		Region 8, Cont. vs. Intra. pressure	n.s.	0.6253
		Region 9, Cont. vs. Intra. pressure	n.s.	0.3821
		Region 10, Cont. vs. Intra. pressure	n.s.	0.8026
	Descriptive	Control 20 min, 1 h, 4 h, n = 27, 36, 30 branches (3 independent experiments)<br>Intra. pressure 20 min, 1 h, 4 h, n = 27, 25, 28 branches (3 independent experiments)		
Fig. 4g	Two-way ANOVA followed by Tukey's test	Day1, 0 μM vs. 30 μM	**	0.0034
		Day1, 0 μM vs. 300 μM	**	<0.0001
		Day2, 0 μM vs. 30 μM	**	<0.0001
		Day2, 0 μM vs. 300 μM	**	<0.0001
	Descriptive	0 μM, n = 60 branches (3 independent experiments)<br>30 μM, n = 60 branches (3 independent experiments)<br>30 μM, n = 75 branches (3 independent experiments)		
Fig. 6b	Two-way ANOVA followed by Tukey's test	siRNA set1: Cont. vs. CIP4	**	<0.0001
		siRNA set1: Cont. vs. TOCA1	**	<0.0001
		siRNA set1: Cont. vs. Both	**	<0.0001
		siRNA set1: CIP4 vs. Both	**	<0.0001
		siRNA set1: TOCA1 vs.Both	**	<0.0001
		siRNA set2: Cont. vs. CIP4	**	<0.0001
		siRNA set2: Cont. vs. TOCA1	**	0.0058
		siRNA set2: Cont. vs. Both	**	<0.0001
		siRNA set2: CIP4 vs. Both	**	<0.0001
		siRNA set2: TOCA1 vs.Both	**	<0.0001
	Descriptive	75 branches for each group (3 independent experiments)		
Fig. 6d	Steel-Dwass test.	siRNA set1: Myr-EGFP, Cont. vs. Myr-EGFP, Both	**	<0.0001
		siRNA set1: Myr-EGFP, Both vs. EGFP-TOCA1, Cont.	**	<0.0001
		siRNA set1: Myr-EGFP, Both vs. EGFP-TOCA1, Both	**	<0.0001

		siRNA set1: Myr-EGFP, Cont. vs. EGFP-TOCA1, Cont.	**	0.0040
		siRNA set1: Myr-EGFP, Cont. vs. EGFP-TOCA1, Both	*	0.0145
		siRNA set2: Myr-EGFP, Cont. vs. Myr-EGFP, Both	**	<0.0001
		siRNA set2: Myr-EGFP, Both vs. EGFP-TOCA1, Cont.	**	<0.0001
		siRNA set2: Myr-EGFP, Both vs. EGFP-TOCA1, Both	**	<0.0001
		siRNA set2: Myr-EGFP, Cont. vs. EGFP-TOCA1, Cont.	n.s.	0.6384
		siRNA set2: Myr-EGFP, Cont. vs. EGFP-TOCA1, Both	n.s.	0.8273
	Descriptive	120 branches for each group (4 independent experiments)		
Fig. 7e	Two-sided Mann-Whitney U test	Myr-EGFP		
		Region 1, Cont. siRNA vs. CIP4/TOCA1 siRNA	n.s.	0.2819
		Region 2, Cont. siRNA vs. CIP4/TOCA1 siRNA	*	0.0418
		Region 3, Cont. siRNA vs. CIP4/TOCA1 siRNA	**	0.0003
		Region 4, Cont. siRNA vs. CIP4/TOCA1 siRNA	n.s.	0.0734
		Region 5, Cont. siRNA vs. CIP4/TOCA1 siRNA	n.s.	0.1033
		Region 6, Cont. siRNA vs. CIP4/TOCA1 siRNA	**	0.003
		Region 7, Cont. siRNA vs. CIP4/TOCA1 siRNA	n.s.	0.4177
		Region 8, Cont. siRNA vs. CIP4/TOCA1 siRNA	**	0.0019
		Region 9, Cont. siRNA vs. CIP4/TOCA1 siRNA	n.s.	0.5516
		Region 10, Cont. siRNA vs. CIP4/TOCA1 siRNA	n.s.	0.6622
		EGFP-TOCA1		
		Region 1, Cont. siRNA vs. CIP4/TOCA1 siRNA	n.s.	0.2479
		Region 2, Cont. siRNA vs. CIP4/TOCA1 siRNA	n.s.	0.9205
		Region 3, Cont. siRNA vs. CIP4/TOCA1 siRNA	n.s.	0.1538
		Region 4, Cont. siRNA vs. CIP4/TOCA1 siRNA	n.s.	0.8317
		Region 5, Cont. siRNA vs. CIP4/TOCA1 siRNA	n.s.	0.0557
		Region 6, Cont. siRNA vs. CIP4/TOCA1 siRNA	n.s.	0.5441
		Region 7, Cont. siRNA vs. CIP4/TOCA1 siRNA	**	0.0004
		Region 8, Cont. siRNA vs. CIP4/TOCA1 siRNA	n.s.	0.3728
		Region 9, Cont. siRNA vs. CIP4/TOCA1 siRNA	n.s.	0.566
		Region 10, Cont. siRNA vs. CIP4/TOCA1 siRNA	n.s.	0.1046
	Descriptive	Myr-EGFP, Cont., n = 18 branches (3 independent experiments)<br>Myr-EGFP, CIP/TOCA1, n = 18 branches (3 independent experiments)<br>EGFP-TPOCA1, Cont., n = 19 branches (3 independent experiments)<br>Myr-EGFP, CIP/TOCA1, n = 20 branches (3 independent experiments)		
Fig. 7f	Two-sided Mann-Whitney U test	Region 1, Cont. siRNA vs. CIP4/TOCA1 siRNA	n.s.	0.0688
		Region 2, Cont. siRNA vs. CIP4/TOCA1 siRNA	n.s.	0.6625
		Region 3, Cont. siRNA vs. CIP4/TOCA1 siRNA	n.s.	0.2156
		Region 4, Cont. siRNA vs. CIP4/TOCA1 siRNA	n.s.	0.4293
		Region 5, Cont. siRNA vs. CIP4/TOCA1 siRNA	n.s.	0.5492
		Region 6, Cont. siRNA vs. CIP4/TOCA1 siRNA	n.s.	0.399
		Region 7, Cont. siRNA vs. CIP4/TOCA1 siRNA	n.s.	0.2901
		Region 8, Cont. siRNA vs. CIP4/TOCA1 siRNA	n.s.	0.8325
		Region 9, Cont. siRNA vs. CIP4/TOCA1 siRNA	n.s.	0.2914
		Region 10, Cont. siRNA vs. CIP4/TOCA1 siRNA	n.s.	0.7995
	Descriptive	Myr-EGFP, Cont., n = 18 branches (3 independent experiments)<br>Myr-EGFP, CIP/TOCA1, n = 18 branches (3 independent experiments)<br>EGFP-TPOCA1, Cont., n = 19 branches (3 independent experiments)		

		Myr-EGFP, CIP/TOCA1, n = 20 branches (3 independent experiments)		
Fig. 7j	Two-way ANOVA followed by Tukey's test	Day1, 0 μM vs. 3 μM	**	0.0035
		Day1, 0 μM vs. 10 μM	**	<0.0001
		Day2, 0 μM vs. 3 μM	**	<0.0001
		Day2, 0 μM vs. 10 μM	**	<0.0001
	Descriptive	0 μM, n = 75 branches (4 independent experiments)<br>3 μM, n = 75 branches (4 independent experiments)<br>10 μM, n = 75 branches (4 independent experiments)		
Fig. 7l	Two-sided Mann-Whitney U test	Actin occupancy		
		Region 1, Cont. vs. Wiskostatin	n.s.	0.784
		Region 2, Cont. vs. Wiskostatin	n.s.	0.1489
		Region 3, Cont. vs. Wiskostatin	**	0.008
		Region 4, Cont. vs. Wiskostatin	n.s.	0.3619
		Region 5, Cont. vs. Wiskostatin	n.s.	0.1073
		Region 6, Cont. vs. Wiskostatin	n.s.	0.1594
		Region 7, Cont. vs. Wiskostatin	*	0.0144
		Region 8, Cont. vs. Wiskostatin	**	0.0045
		Region 9, Cont. vs. Wiskostatin	**	0.005
		Region 10, Cont. vs. Wiskostatin	**	0.0001
		Arp2/3 complexes		
		Region 1, Cont. vs. Wiskostatin	n.s.	0.9517
		Region 2, Cont. vs. Wiskostatin	n.s.	0.5976
		Region 3, Cont. vs. Wiskostatin	**	0.0047
		Region 4, Cont. vs. Wiskostatin	**	0.002
		Region 5, Cont. vs. Wiskostatin	**	0.0085
		Region 6, Cont. vs. Wiskostatin	n.s.	0.1047
		Region 7, Cont. vs. Wiskostatin	n.s.	0.1593
		Region 8, Cont. vs. Wiskostatin	n.s.	0.0613
		Region 9, Cont. vs. Wiskostatin	n.s.	0.8685
		Region 10, Cont. vs. Wiskostatin	n.s.	0.122
		Relative area		
		Region 1, Cont. vs. Wiskostatin	n.s.	0.574
		Region 2, Cont. vs. Wiskostatin	n.s.	0.1045
		Region 3, Cont. vs. Wiskostatin	*	0.0102
		Region 4, Cont. vs. Wiskostatin	*	0.0102
		Region 5, Cont. vs. Wiskostatin	**	0.0015
		Region 6, Cont. vs. Wiskostatin	**	0.006
		Region 7, Cont. vs. Wiskostatin	*	0.046
		Region 8, Cont. vs. Wiskostatin	n.s.	0.1248
		Region 9, Cont. vs. Wiskostatin	n.s.	0.3384
		Region 10, Cont. vs. Wiskostatin	n.s.	0.3127
	Descriptive	Cont., n = 19 branches (3 independent experiments)<br>Wiskostatin, n = 18 branches (3 independent experiments)		
Fig. 8f	Two-sided Mann-Whitney U test	CIP4 clusters		
		Region 1, Cont. vs. Intra. pressure	n.s.	0.1023
		Region 2, Cont. vs. Intra. pressure	*	0.0108
		Region 3, Cont. vs. Intra. pressure	**	0.0001
		Region 4, Cont. vs. Intra. pressure	**	0.0002

		Region 5, Cont. vs. Intra. pressure	**	<0.0001
		Region 6, Cont. vs. Intra. pressure	**	0.0004
		Region 7, Cont. vs. Intra. pressure	n.s.	0.937
		Region 8, Cont. vs. Intra. pressure	n.s.	0.0946
		Region 9, Cont. vs. Intra. pressure	n.s.	0.1088
		Region 10, Cont. vs. Intra. pressure	n.s.	0.1073
		Arp2/3 complexes		
		Region 1, Cont. vs. Intra. pressure	n.s.	0.2845
		Region 2, Cont. vs. Intra. pressure	**	0.0006
		Region 3, Cont. vs. Intra. pressure	**	<0.0001
		Region 4, Cont. vs. Intra. pressure	**	<0.0001
		Region 5, Cont. vs. Intra. pressure	**	<0.0001
		Region 6, Cont. vs. Intra. pressure	**	0.0018
		Region 7, Cont. vs. Intra. pressure	n.s.	0.2845
		Region 8, Cont. vs. Intra. pressure	*	0.0436
		Region 9, Cont. vs. Intra. pressure	n.s.	0.1846
		Region 10, Cont. vs. Intra. pressure	**	0.0035
		Colocalization index		
		Region 1, Cont. vs. Intra. pressure	n.s.	0.2745
		Region 2, Cont. vs. Intra. pressure	n.s.	0.1958
		Region 3, Cont. vs. Intra. pressure	n.s.	0.1476
		Region 4, Cont. vs. Intra. pressure	**	0.0025
		Region 5, Cont. vs. Intra. pressure	**	0.0038
		Region 6, Cont. vs. Intra. pressure	*	0.0148
		Region 7, Cont. vs. Intra. pressure	n.s.	0.9451
		Region 8, Cont. vs. Intra. pressure	n.s.	0.5174
		Region 9, Cont. vs. Intra. pressure	n.s.	0.7514
		Region 10, Cont. vs. Intra. pressure	n.s.	0.0520
	Descriptive	Cont., n = 23 branches (3 independent experiments)<br>Intra. pressure, n = 19 branches (3 independent experiments)		
Fig. 8h	Two-sided Mann-Whitney U test	CIP4 clusters		
		Region 1, Isotonic vs. Hypotonic	*	0.0102
		Region 2, Isotonic vs. Hypotonic	**	0.0002
		Region 3, Isotonic vs. Hypotonic	**	0.0003
		Region 4, Isotonic vs. Hypotonic	*	0.0124
		Region 5, Isotonic vs. Hypotonic	**	0.0011
		Region 6, Isotonic vs. Hypotonic	**	0.0049
		Region 7, Isotonic vs. Hypotonic	*	0.0441
		Region 8, Isotonic vs. Hypotonic	**	0.0030
		Region 9, Isotonic vs. Hypotonic	*	0.0133
		Region 10, Isotonic vs. Hypotonic	**	0.0028
		Arp2/3 complexes		
		Region 1, Isotonic vs. Hypotonic	n.s.	0.0995
		Region 2, Isotonic vs. Hypotonic	**	0.0002
		Region 3, Isotonic vs. Hypotonic	**	0.0005
		Region 4, Isotonic vs. Hypotonic	*	0.0230
		Region 5, Isotonic vs. Hypotonic	n.s.	0.0855
		Region 6, Isotonic vs. Hypotonic	n.s.	0.0985

		Region 7, Isotonic vs. Hypotonic	n.s.	0.9023
		Region 8, Isotonic vs. Hypotonic	n.s.	0.1568
		Region 9, Isotonic vs. Hypotonic	n.s.	0.3039
		Region 10, Isotonic vs. Hypotonic	n.s.	0.2913
		Colocalization index		
		Region 1, Isotonic vs. Hypotonic	**	0.0047
		Region 2, Isotonic vs. Hypotonic	**	<0.0001
		Region 3, Isotonic vs. Hypotonic	**	<0.0001
		Region 4, Isotonic vs. Hypotonic	**	0.0013
		Region 5, Isotonic vs. Hypotonic	*	0.0178
		Region 6, Isotonic vs. Hypotonic	n.s.	0.3038
		Region 7, Isotonic vs. Hypotonic	n.s.	0.1337
		Region 8, Isotonic vs. Hypotonic	n.s.	0.8055
		Region 9, Isotonic vs. Hypotonic	n.s.	0.6066
		Region 10, Isotonic vs. Hypotonic	n.s.	0.1337
	Descriptive	Cont., n = 20 branches (3 independent experiments)<br>Intra. pressure, n = 22 branches (3 independent experiments)		
Supplementary Fig. 10b	Two-sided Mann-Whitney U test	20 min		
		Region 1, Cont. vs. Intra. pressure	*	0.0418
		Region 2, Cont. vs. Intra. pressure	**	0.0027
		Region 3, Cont. vs. Intra. pressure	**	<0.0001
		Region 4, Cont. vs. Intra. pressure	**	0.0002
		Region 5, Cont. vs. Intra. pressure	*	0.0384
		Region 6, Cont. vs. Intra. pressure	n.s.	0.2687
		Region 7, Cont. vs. Intra. pressure	n.s.	0.7936
		Region 8, Cont. vs. Intra. pressure	n.s.	0.7804
		Region 9, Cont. vs. Intra. pressure	n.s.	0.8874
		Region 10, Cont. vs. Intra. pressure	n.s.	0.8739
		1 h		
		Region 1, Cont. vs. Intra. pressure	n.s.	0.3603
		Region 2, Cont. vs. Intra. pressure	*	0.0320
		Region 3, Cont. vs. Intra. pressure	**	0.0002
		Region 4, Cont. vs. Intra. pressure	**	0.0013
		Region 5, Cont. vs. Intra. pressure	n.s.	0.0519
		Region 6, Cont. vs. Intra. pressure	n.s.	0.2665
		Region 7, Cont. vs. Intra. pressure	n.s.	0.0527
		Region 8, Cont. vs. Intra. pressure	n.s.	0.3976
		Region 9, Cont. vs. Intra. pressure	n.s.	0.9787
		Region 10, Cont. vs. Intra. pressure	n.s.	0.6118
		4 h		
		Region 1, Cont. vs. Intra. pressure	*	0.0124
		Region 2, Cont. vs. Intra. pressure	**	0.0005
		Region 3, Cont. vs. Intra. pressure	**	<0.0001
		Region 4, Cont. vs. Intra. pressure	**	<0.0001
		Region 5, Cont. vs. Intra. pressure	**	0.0001
		Region 6, Cont. vs. Intra. pressure	**	0.0010
		Region 7, Cont. vs. Intra. pressure	**	0.0029
		Region 8, Cont. vs. Intra. pressure	*	0.0168

		Region 9, Cont. vs. Intra. pressure	n.s.	0.112
		Region 10, Cont. vs. Intra. pressure	n.s.	0.3181
	Descriptive	Control 20 min, 1 h, 4 h, n = 27, 36, 30 branches (3 independent experiments)<br>Intra. pressure 20 min, 1 h, 4 h, n = 27, 25, 28 branches (3 independent experiments)		
Supplementary Fig. 10d	Steel-Dwass test	Region 1, Cont. vs. Intra. pressure	n.s.	0.2394
		Region 2, Cont. vs. Intra. pressure	**	0.0001
		Region 3, Cont. vs. Intra. pressure	**	0.0001
		Region 4, Cont. vs. Intra. pressure	**	0.0008
		Region 5, Cont. vs. Intra. pressure	n.s.	0.1177
		Region 6, Cont. vs. Intra. pressure	n.s.	0.4320
		Region 7, Cont. vs. Intra. pressure	n.s.	0.1217
		Region 8, Cont. vs. Intra. pressure	n.s.	0.2954
		Region 9, Cont. vs. Intra. pressure	n.s.	0.9744
		Region 10, Cont. vs. Intra. pressure	n.s.	0.6425
		Region 1, Extra. pressure vs. Intra. pressure	n.s.	0.5028
		Region 2, Extra. pressure vs. Intra. pressure	n.s.	0.0813
		Region 3, Extra. pressure vs. Intra. pressure	**	0.0034
		Region 4, Extra. pressure vs. Intra. pressure	**	0.0002
		Region 5, Extra. pressure vs. Intra. pressure	n.s.	0.3137
		Region 6, Extra. pressure vs. Intra. pressure	n.s.	0.1130
		Region 7, Extra. pressure vs. Intra. pressure	*	0.0115
		Region 8, Extra. pressure vs. Intra. pressure	n.s.	0.8342
		Region 9, Extra. pressure vs. Intra. pressure	n.s.	0.9735
		Region 10, Extra. pressure vs. Intra. pressure	n.s.	0.9991
		Region 1, Intra./Extra. pressure vs. Intra. pressure	n.s.	0.2797
		Region 2, Intra./Extra. pressure vs. Intra. pressure	n.s.	0.0856
		Region 3, Intra./Extra. pressure vs. Intra. pressure	**	0.0054
		Region 4, Intra./Extra. pressure vs. Intra. pressure	**	<0.0001
		Region 5, Intra./Extra. pressure vs. Intra. pressure	*	0.0147
		Region 6, Intra./Extra. pressure vs. Intra. pressure	n.s.	0.0924
		Region 7, Intra./Extra. pressure vs. Intra. pressure	n.s.	0.1588
		Region 8, Intra./Extra. pressure vs. Intra. pressure	n.s.	0.2293
		Region 9, Intra./Extra. pressure vs. Intra. pressure	n.s.	0.7362
		Region 10, Intra./Extra. pressure vs. Intra. pressure	n.s.	0.8150
	Descriptive	Cont., n = 21 branches (3 independent experiments)<br>Intra. pressure, n = 17 branches (3 independent experiments)<br>Extra. pressure, n = 18 branches (3 independent experiments)<br>Intra./Extra. Pressure, n = 23 branches (3 independent experiments)		
Supplementary Fig. 20b	Two-sided Mann-Whitney U test	EGFP-TOCA1 clusters		
		Region 1, Cont. vs. Intra. pressure	**	<0.0001
		Region 2, Cont. vs. Intra. pressure	*	0.0453
		Region 3, Cont. vs. Intra. pressure	**	<0.0001
		Region 4, Cont. vs. Intra. pressure	**	0.0002
		Region 5, Cont. vs. Intra. pressure	n.s.	0.1254
		Region 6, Cont. vs. Intra. pressure	*	0.0468
		Region 7, Cont. vs. Intra. pressure	n.s.	0.4788
		Region 8, Cont. vs. Intra. pressure	*	0.0320
		Region 9, Cont. vs. Intra. pressure	n.s.	0.1566

		Region 10, Cont. vs. Intra. pressure	n.s.	0.1001
		Arp2/3 complexes		
		Region 1, Cont. vs. Intra. pressure	**	0.0008
		Region 2, Cont. vs. Intra. pressure	**	0.0036
		Region 3, Cont. vs. Intra. pressure	**	0.0003
		Region 4, Cont. vs. Intra. pressure	**	0.0010
		Region 5, Cont. vs. Intra. pressure	*	0.0137
		Region 6, Cont. vs. Intra. pressure	**	0.0074
		Region 7, Cont. vs. Intra. pressure	n.s.	0.2869
		Region 8, Cont. vs. Intra. pressure	n.s.	0.0803
		Region 9, Cont. vs. Intra. pressure	n.s.	0.5541
		Region 10, Cont. vs. Intra. pressure	n.s.	0.718
		Colocalization index		
		Region 1, Cont. vs. Intra. pressure	*	0.0109
		Region 2, Cont. vs. Intra. pressure	*	0.0308
		Region 3, Cont. vs. Intra. pressure	**	0.0024
		Region 4, Cont. vs. Intra. pressure	**	0.0002
		Region 5, Cont. vs. Intra. pressure	**	0.0076
		Region 6, Cont. vs. Intra. pressure	*	0.0468
		Region 7, Cont. vs. Intra. pressure	n.s.	0.2939
		Region 8, Cont. vs. Intra. pressure	n.s.	1
		Region 9, Cont. vs. Intra. pressure	n.s.	0.7934
		Region 10, Cont. vs. Intra. pressure	**	0.0086
	Descriptive	Control, n = 22 branches (3 independent experiments)<br>Intra. Pressure, n = 22 branches (3 independent experiments)		
Supplementary Fig. 20c	Two-sided Mann-Whitney U test	EGFP-CIP4 clusters		
		Region 1, Cont. vs. Intra. pressure	*	0.0470
		Region 2, Cont. vs. Intra. pressure	**	0.0025
		Region 3, Cont. vs. Intra. pressure	**	0.0005
		Region 4, Cont. vs. Intra. pressure	**	0.0013
		Region 5, Cont. vs. Intra. pressure	**	0.0058
		Region 6, Cont. vs. Intra. pressure	*	0.0151
		Region 7, Cont. vs. Intra. pressure	*	0.0488
		Region 8, Cont. vs. Intra. pressure	n.s.	0.1129
		Region 9, Cont. vs. Intra. pressure	n.s.	0.6101
		Region 10, Cont. vs. Intra. pressure	n.s.	0.5466
		Arp2/3 complexes		
		Region 1, Cont. vs. Intra. pressure	*	0.0360
		Region 2, Cont. vs. Intra. pressure	**	0.0001
		Region 3, Cont. vs. Intra. pressure	**	0.0000
		Region 4, Cont. vs. Intra. pressure	**	0.0046
		Region 5, Cont. vs. Intra. pressure	**	0.0031
		Region 6, Cont. vs. Intra. pressure	**	0.0000
		Region 7, Cont. vs. Intra. pressure	**	0.0012
		Region 8, Cont. vs. Intra. pressure	**	0.0022
		Region 9, Cont. vs. Intra. pressure	n.s.	0.0787
		Region 10, Cont. vs. Intra. pressure	n.s.	0.7869
		Colocalization index		

		Region 1, Cont. vs. Intra. pressure	*	0.011
		Region 2, Cont. vs. Intra. pressure	**	0.0018
		Region 3, Cont. vs. Intra. pressure	**	<0.0001
		Region 4, Cont. vs. Intra. pressure	**	<0.0001
		Region 5, Cont. vs. Intra. pressure	**	<0.0001
		Region 6, Cont. vs. Intra. pressure	**	0.0020
		Region 7, Cont. vs. Intra. pressure	*	0.0236
		Region 8, Cont. vs. Intra. pressure	n.s.	0.1732
		Region 9, Cont. vs. Intra. pressure	**	0.0025
		Region 10, Cont. vs. Intra. pressure	*	0.0188
	Descriptive	Control, n = 18 branches (3 independent experiments)<br>Intra. Pressure, n = 20 branches (3 independent experiments)		
