# Supplementary material for: Mechanical loading of intraluminal pressure mediates wound angiogenesis by regulating the TOCA family of F-BAR proteins
Source: Nat Commun. 2022 May 12;13:2594. doi: 10.1038/s41467-022-30197-8 (PMC9098626; doi:10.1038/s41467-022-30197-8)
Supplement: Supplementary file 3 — Description of Additional Supplementary Files [file 41467_2022_30197_MOESM3_ESM.docx]

**Description of Additional Supplementary Files**

**File Name: Supplementary Movie 1**

**Description:** Long-term confocal time-lapse imaging of wound angiogenesis in the injured skin of the *Tg(kdrl:EGFP)* adult zebrafish from 1.3-10.2 dpi. Elapsed time (hr:min:sec:msec) is shown in the upper right corner. Scale bar, 50 μm. See also Supplementary Fig. 1a.

**File Name:** **Supplementary Movie 2**

**Description:** Time-lapse confocal imaging of wound angiogenesis in the injured skin of the *Tg(kdrl:EGFP)* adult zebrafish. Images were obtained every 6 h from 0.7-96.7 hpi. Elapsed time (hr:min:sec:msec) is shown in the upper right corner. Scale bar, 50 μm. See also Fig. 1a.

**File Name:** **Supplementary Movie 3**

**Description:** Time-lapse confocal imaging of reparative process of a single injured capillary in the trunk of the *Tg(kdrl:EGFP)* adult zebrafish. The injured portion was approximately 560 μm in length. Images were obtained every 12 h from 0.6-72 hpi. The vessel located downstream from blood flow is the one elongating from the upper side. Elapsed time (hr:min:sec:msec) is shown in the upper right corner. Scale bar, 50 μm. See also Fig. 1b.

**File Name:** **Supplementary Movie 4**

**Description:** Time-lapse confocal imaging of reparative process of a single injured capillary in the trunk of the *Tg(kdrl:EGFP)* adult zebrafish. In contrast to the experiments described in Supplementary Movie 3, only a small portion of the capillary (approximately 260 μm in length) was injured in this experiment. Images were obtained every 6 h from 0.4-30.4 hpi. The vessel located downstream from blood flow is the one elongating from the upper side. Elapsed time (hr:min:sec:msec) is shown in the upper right corner. Scale bar, 50 μm. See also Supplementary Fig. 1b.

**File Name:** **Supplementary Movie 5**

**Description:** Time-lapse confocal imaging of angiogenesis in an injured arterial ISV of the *Tg(fli1a:EGFP)* zebrafish larva at 3 dpf. Images were obtained every 15 min from 0.4-11.1 hpi. Lateral view, anterior to the left. Elapsed time (hr:min:sec:msec) is shown in the upper right corner. Scale bar, 50 μm. See also Fig. 1d.

**File Name:** **Supplementary Movie 6**

**Description:** Time-lapse confocal imaging of angiogenesis in an injured venous ISV of the *Tg(fli1a:EGFP)* zebrafish larva at 3dpf. Images were obtained every 15 min from 0.6-11.1 hpi. Lateral view, anterior to the left. Elapsed time (hr:min:sec:msec) is shown in the upper right corner. Scale bar, 50 μm. See also Supplementary Fig. 1c.

**File Name:** **Supplementary Movie 7**

**Description:** Time-lapse confocal imaging of an injured aISV in 3 dpf *Tg(fli1a:Myr-EGFP)* larva intravascularly injected with PEGylated fluorescent microspheres (PEGylated FM) and Qdots (Qdot 705). Image acquisition was started at 2.7 hpi and 37 min after the injection. Images were obtained at a rate of 73 frames per min for 1 min. Lateral view, anterior to the left. Merged image of EGFP (green), PEGylated FM (magenta) and Qdot (blue). Elapsed time (hr:min:sec:msec) is shown in the upper right corner. Note that no PEGylated FM entered the upstream and downstream injured vessels during this time period. Scale bar, 10 μm. See also Fig. 2a (0-1.0 min).

**File Name:** **Supplementary Movie 8**

**Description:** Time-lapse confocal imaging of an injured aISV in same zebrafish larva as one shown in Supplementary Movie 7. Image acquisition was started 13 min after imaging for Supplementary Movie 7. Lateral view, anterior to the left. Merged image of EGFP (green), PEGylated FM (magenta) and Qdot (blue). Elapsed time (hr:min:sec:msec) is shown in the upper right corner. Note that some PEGylated FM, which ended up in upstream injured vessels, showed Brownian motion-like movement when entering upstream injured vessels by chance. Scale bar, 10 μm. See also Fig. 2a (14.0-15.0 min).

**File Name:** **Supplementary Movie 9**

**Description:** Time-lapse confocal imaging of dorsal aorta and aISV in trunk region of 3 dpf *Tg(fli1a:Myr-EGFP)* larva intravascularly injected with PEGylated fluorescent microspheres (PEGylated FM). Images were obtained at a rate of 84 frames per min for 1 min. Lateral view, anterior to the left. Merged image of EGFP (green) and PEGylated FM (magenta). Elapsed time (hr:min:sec:msec) is shown in the upper right corner. Scale bar, 10 μm. See also Fig. 2b.

**File Name:** **Supplementary Movie 10**

**Description:** Time-lapse confocal imaging of dorsal aorta and base of aISV in trunk region of 3 dpf *Tg(fli1a:Myr-EGFP)* larva intravascularly injected with PEGylated FM. Images were obtained at a rate of 88 frames per min for 1 min. Lateral view, anterior to the left. Merged image of EGFP (green) and PEGylated FM (magenta). Elapsed time (hr:min:sec:msec) is shown in the upper right corner. Scale bar, 10 μm. See also Supplementary Fig. 4d.

**File Name:** **Supplementary Movie 11**

**Description:** Timelapse confocal imaging of an injured aISV in 3 dpf *Tg(fli1a:Myr-EGFP)* larva intravascularly injected with Qdots (Qdot 655). Before imaging, the larva underwent experimental procedure as described in the bottom of Supplementary Fig. 4e. Initially, a single aISV was injured by laser ablation, and subsequently the heartbeat was arrested by treatment with high concentration of tricaine (0.12-0.13% in E3 imaging medium). After injection of Qdots into the pericardial cavity, the larva was washed with E3 imaging medium to remove tricaine and immediately subjected to timelapse imaging before blood flow started. Images were obtained every 20 s for 10 min. Lateral view, anterior to the left. Left, merged images of EGFP (green) and Qdot (blue); right, Qdot images. Elapsed time (hr:min:sec:msec) is shown in the upper right corner. Note that the dorsal aorta was quickly filled with Qdots when blood flow started, whereas the Qdots moved only gradually from the dorsal aorta to the tip of the upstream injured aISV (approximately 0.3 μm/s). Scale bar, 10 μm. See also Supplementary Fig. 4e.

**File Name:** **Supplementary Movie 12**

**Description:** Time-lapse confocal imaging of reparative process of a single injured capillary in which intraluminal pressure (IP) of the upstream injured vessel was relieved by cutting the more upstream site in the skin of *Tg(kdrl:EGFP)* adult zebrafish. This movie was created from sequential still images obtained at 1.2, 18, 29, 42, and 54 hpi. The injured upstream vessel not loaded with IP is the one elongating from the upper side. Scale bar, 50 μm. See also Fig. 2c.

**File Name:** **Supplementary Movie 13**

**Description:** Acute changes in vascular morphology of an on-chip angiogenic branch after IP loading. DIC images were taken at 10 frames per second for 20 seconds. See also Supplementary Fig. 7b.

**File Name:** **Supplementary Movie 14**

**Description:** Acute changes in vascular morphology of an on-chip angiogenic branch after extraluminal pressure (EP) loading. DIC images were taken at 10 frames per second for 20 seconds. See also Supplementary Fig. 7d.

**File Name:** **Supplementary Movie 15**

**Description:** Acute changes in vascular morphology of an EP-loaded on-chip angiogenic branch after additional IP loading. DIC images were taken at 10 frames per second for 20 seconds. See also Supplementary Fig. 7d.

**File Name:** **Supplementary Movie 16**

**Description:** Acute changes in vascular morphology of IP-loaded on-chip angiogenic branch after release of pressure. DIC images were taken at 10 frames per second for 20 seconds. See also Fig. 3j.

**File Name:** **Supplementary Movie 17**

**Description:** Dynamics of an IP-loaded on-chip angiogenic branch after release of pressure. Time-lapse DIC images were taken every 15 minutes over 55 hours. Movie is shown at a frame rate of 15 frames per second. See also Fig. 3j.

**File Name:** **Supplementary Movie 18**

**Description:** Dynamics of localization of EYFP-Golgi in an EC of an on-chip angiogenic branch comprised of mosaic HUVECs with one expressing EYFP-Golgi before IP loading. Time-lapse fluorescent and DIC images were taken every 15 minutes over 10 hours. Blue color indicates nuclei with Hoechst staining. Red dots indicate the trajectory of the tip of elongating angiogenic branches. This movie is shown at a rate of 15 frames per second. See also Supplementary Fig. 9c.

**File Name:** **Supplementary Movie 19**

**Description:** Dynamics of localization of EYFP-Golgi in an EC of an on-chip angiogenic branch comprised of mosaic HUVECs with one expressing EYFP-Golgi after IP loading. Time-lapse fluorescent and DIC images were taken every 15 minutes over 8 hours. Blue color indicates nuclei with Hoechst staining. Blue dots indicate the trajectory of the tip of retracting angiogenic branches. This movie is shown at a rate of 15 frames per second. See also Supplementary Fig. 9c.

**File Name:** **Supplementary Movie 20**

**Description:** Time-lapse confocal imaging of angiogenesis in an injured arterial ISV of the *Tg(fli1a:mCherry);(fli1a:Golgi-EYFP)* larval zebrafish. Images were obtained every 20 min from 0.8-7.1 hpi. Magenta, mCherry; green, Golgi-EYFP. Lateral view, anterior to the left. Elapsed time (hr:min:sec:msec) is shown in the upper right corner. Scale bar, 10 μm. See also Fig. 5a.

**File Name:** **Supplementary Movie 21**

**Description:** Time-lapse confocal imaging of angiogenesis in an injured aISV of 3 dpf *Tg(fli1a:mCherry);(fli1a:Golgi-EYFP)* larva injected with low-dose *aplnrb* dgRNA:Cas9 RNP complex. Images were obtained every 20 min from 0.5-6.5 hpi. Magenta, mCherry; green, Golgi-EYFP. Lateral view, anterior to the left. Elapsed time (hr:min:sec:msec) is shown in the upper right corner. Note that the EC in the tip of upstream injured aISV positioned its Golgi apparatus ahead of the nucleus toward the vessel elongation direction at the beginning of time-lapse imaging. Scale bar, 10 μm. See also Supplementary Fig. 12e.

**File Name:** **Supplementary Movie 22**

**Description:** Time-lapse confocal imaging of angiogenesis in an injured arterial ISV of the *Tg(fli1a:Lifeact-mCherry)* zebrafish larva at 3 dpf. Images were obtained every 50 min from 1.1-8.6 hpi. White indicates actin filaments. Lateral view, anterior to the left. Elapsed time (hr:min:sec:msec) is shown in the upper right corner. Scale bar, 10 μm. See also Fig. 5d.

**File Name:** **Supplementary Movie 23**

**Description:** Time-lapse confocal imaging of ISV formation in the *Tg(fli1a:EGFP-ARPC4);(fli1a:lifeact-mCherry)* zebrafish embryo. The recording started at 23 hpf. Images were obtained every 10 min for 6.5 h. Left, EGFP-ARPC4; middle, Lifeact-mCherry; right, the merged images of EGFP-ARPC4 and Lifeact-mCherry. Lateral view, anterior to the left. Elapsed time (hr:min:sec:msec) is shown in the upper left corner of each image. Scale bar, 10 μm. See also Supplementary Fig. 13a.

**File Name:** **Supplementary Movie 24**

**Description:** Time-lapse confocal imaging of ISV formation in the *Tg(fli1a:Lifeact-mCherry);(fli1a:Myr-EGFP)* zebrafish embryos treated with vehicle (left) or 200 μM CK-666 (right). The Lifeact-mCherry signal (actin filaments) is shown. The recording started at 24 hpf. Images were obtained every 10 min for 3 h. Lateral view, anterior to the left. Elapsed time (hr:min:sec:msec) is shown in the upper right corner. Scale bar, 10 μm. See also Supplementary Fig. 13b.

**File Name:** **Supplementary Movie 25**

**Description:** Time-lapse confocal imaging of angiogenesis in an injured arterial ISV of the *Tg(fli1a:EGFP-ARPC4);(fli1a:Lifeact-mCherry)* zebrafish larva at 3 dpf. Images were obtained every 15 min from 0.7-6.7 hpi. Left, EGFP-ARPC4; middle, Lifeact-mCherry; right, the merged images of EGFP-ARPC4 and Lifeact-mCherry. Lateral view, anterior to the left. Elapsed time (hr:min:sec:msec) is shown in the upper left corner. Scale bar, 10 μm. See also Fig. 5f.

**File Name:** **Supplementary Movie 26**

**Description:** Time-lapse confocal imaging of ISV formation in the *Tg(fli1a:EGFP-toca1);(fli1a:lifeact-mCherry)* zebrafish embryo. The recording started at 23 hpf. Images were obtained every 15 min for 6.5 h. Left, EGFP-Toca1; middle, Lifeact-mCherry; right, the merged images of EGFP-Toca1 and Lifeact-mCherry. Lateral view, anterior to the left. Elapsed time (hr:min:sec:msec) is shown in the upper left corner of each image. Scale bar, 10 μm. See also Fig. 7c.

**File Name:** **Supplementary Movie 27**

**Description:** Time-lapse confocal imaging of angiogenesis in an injured arterial ISV of the *Tg(fli1a:EGFP-toca1);(fli1a:Lifeact-mCherry)* larval zebrafish. Images were obtained every 15 min from 0.6-10.2 hpi. Left, EGFP-toca1; middle, Lifeact-mCherry; right, the merged images of EGFP-Toca1 and Lifeact-mCherry. Lateral view, anterior to the left. Elapsed time (hr:min:sec:msec) is shown in the upper left corner of each image. Scale bar, 10 μm. See also Fig. 9a.
