# Supplementary material for: Mechanical loading of intraluminal pressure mediates wound angiogenesis by regulating the TOCA family of F-BAR proteins
Source: Nat Commun. 2022 May 12;13:2594. doi: 10.1038/s41467-022-30197-8 (PMC9098626; doi:10.1038/s41467-022-30197-8)
Supplement: Supplementary file 31 — Reporting Summary [file 41467_2022_30197_MOESM31_ESM.pdf]

nature portfolio

Corresponding author(s): Shigetomo Fukuhara and Koichi Nishiyama

Last updated by author(s): Mar 2, 2022

Reporting Summary

Nature Portfolio wishes to improve the reproducibility of the work that we publish. This form provides structure for consistency and transparency in reporting. For further information on Nature Portfolio policies, see our Editorial Policies and the Editorial Policy Checklist.

Statistics

For all statistical analyses, confirm that the following items are present in the figure legend, table legend, main text, or Methods section.

n/a	Confirmed	
☐	☒	The exact sample size (*n*) for each experimental group/condition, given as a discrete number and unit of measurement
☐	☒	A statement on whether measurements were taken from distinct samples or whether the same sample was measured repeatedly
☐	☒	The statistical test(s) used AND whether they are one- or two-sided<br>*Only common tests should be described solely by name; describe more complex techniques in the Methods section.*
☒	☐	A description of all covariates tested
☐	☒	A description of any assumptions or corrections, such as tests of normality and adjustment for multiple comparisons
☐	☒	A full description of the statistical parameters including central tendency (e.g. means) or other basic estimates (e.g. regression coefficient) AND variation (e.g. standard deviation) or associated estimates of uncertainty (e.g. confidence intervals)
☐	☒	For null hypothesis testing, the test statistic (e.g. *F*, *t*, *r*) with confidence intervals, effect sizes, degrees of freedom and *P* value noted<br>*Give P values as exact values whenever suitable.*
☒	☐	For Bayesian analysis, information on the choice of priors and Markov chain Monte Carlo settings
☒	☐	For hierarchical and complex designs, identification of the appropriate level for tests and full reporting of outcomes
☒	☐	Estimates of effect sizes (e.g. Cohen's *d*, Pearson's *r*), indicating how they were calculated

Our web collection on statistics for biologists contains articles on many of the points above.

Software and code

Policy information about availability of computer code

Data collection	Image acquisition for in vivo experiments: Fluorescence images were acquired using FLUOVIEW FV1000 and FV1200 microscopes (Olympus) operated with FLUOVIEW FV10ASW software (Olympus), and FLUOVIEW FV3000 (Olympus) and FVMPE-RS microscopes (Olympus) operated with FLUOVIEW FV31S-SW software (Olympus), and A1R MP+ microscope (Nikon) operated with NIS-Elements software (Nikon), as described in detail in the Methods section. Bright field images of zebrafish larvae were captured using SZX16 microscope (Olympus).<br><br>Image acquisition for on-chip angiogenesis assays: All DIC and fluorescent images were obtained by a commercially available fluorescent inverted microscope (IX83, Olympus), equipped with a CMOS camera (ORCA-Flash4.0, Hamamatsu Photonics) operated by MetaMorph operating software version 7.10.1.161 (Molecular device). All confocal images were obtained using a confocal laser-scanning microscope (FluoView FV1200, Olympus), equipped with a GaAsP detector (Olympus) operated by FV10-ASM4.2 software (Olympus) and using a confocal laser-scanning microscope (TCS-SP8, Leica), equipped with a Hybrid Detector (Leica HyD, Leica), operated by LASX version2.0.1.14392 (Leica).<br><br>Image acquisition for in vitro would healing assays: Images were obtained using FLUOVIEW FV3000 microscopes (Olympus).<br><br>Data collection for quantitative PCR analysis: Light Cycler 96 (Roche).<br><br>Data collection for western blot analysis: Chemiluminescence signals were obtained using the ChemiDoc™ Imaging System (Bio-Rad) and LAS4000 (Cytiva). Fluorescence signals were obtained using the Image analyzer ODYSSEY Fc Imaging System (LI-COR Bioscience Systems).
Data analysis	Analysis for in vivo imaging data: Image data obtained from in vivo experiments were analyzed using Volocity 6.3 3D Imaging Analysis software (Quorum Technologies), Fiji/ImagJ software package (http://fiji.sc.), FLUOVIEW FV10ASW and FV31S-SW software (Olympus), and Photoshop CS4 and 2021 software (Adobe), as described in detail in the Methods section.<br><br>Analysis for imaging data obtained from on-chip angiogenesis assays: All image data in in vitro on-chip angiogenesis experiments were processed using Fiji/ImageJ 2.1.0/1.53n for analyses and presentation, as mentioned in Methods. After appropriate image processing, DIC

images were subsequently analyzed for quantification of branch length in on-chip angiogenesis using the Fiji/ImageJ 2.1.0/1.53n (http://fiji.sc.) and confocal images were subjected to quantitative analyses for front-rear polarity of ECs, vascular tip morphology, and localization and co-localization patterns of various molecules (F-actin, Arp2/3 complexes, CIP4 and EGFP-TOCA-1 using Mathematica 12 version 12.0.0.0 (Wolfram).

Analysis for in vitro wound healing assay data: Image data were analyzed using MetaMorph 7.8 Basic software (Molecular Devices), except for preparing representative images by Volocity 6.3 3D Imaging Analysis software (Quorum Technologies).

Statistical analysis: Statistical analyses for in vivo data were carried out using GraphPad Prism 8 software version 8.4.3 (GraphPad Software) or KyPlot 6.0 (KyensLab), while those for in vitro experiments were done using GraphPad Prism 8 software version 8.4.3 or R 3.3.3 (GraphPad Software).

For manuscripts utilizing custom algorithms or software that are central to the research but not yet described in published literature, software must be made available to editors and reviewers. We strongly encourage code deposition in a community repository (e.g. GitHub). See the Nature Portfolio guidelines for submitting code & software for further information.

Data

Policy information about availability of data

All manuscripts must include a data availability statement. This statement should provide the following information, where applicable:

- Accession codes, unique identifiers, or web links for publicly available datasets
- A description of any restrictions on data availability
- For clinical datasets or third party data, please ensure that the statement adheres to our policy

The authors declare that all data supporting the findings of this study are available within the main text and supplementary materials. Raw data to generate all graphs within the Figures and Supplementary Figures and uncropped scans of all blots and gels in the Supplementary Figures are provided as a Source Data File. Source data are provided with this paper.

Field-specific reporting

Please select the one below that is the best fit for your research. If you are not sure, read the appropriate sections before making your selection.

☒ Life sciences ☐ Behavioural & social sciences ☐ Ecological, evolutionary & environmental sciences

For a reference copy of the document with all sections, see nature.com/documents/nr-reporting-summary-flat.pdf

Life sciences study design

All studies must disclose on these points even when the disclosure is negative.

Sample size

In vivo experiments: No statistical method was used to predetermine sample size. Sample size was determined based on our previous studies (doi: 10.1007/s10456-018-09660-y, doi: 10.1016/j.devcel.2014.11.024). Sample sizes are stated in figure legends or in the Results section.

In vitro on-chip angiogenesis assay: No statistical method was used to predetermined the sample size. Sample size for individual quantification experiment was determined by analyzing all branches or cells in each chip or selected branches in each chip through at least 3 independent experiments except for localization analysis of exogeneous EGFP-ARPC4 in stalk cells in Supplementary Fig. 10f, g to sufficiently secure data reproducibility as well as to prevent biased sampling.

In vitro would healing assay: No statistical method was used to predetermine sample size. Sample size for quantification experiments was determined by analyzing four randomly-selected regions in each experiment through 2 independent experiments to sufficiently secure data reproducibility as well as to prevent biased sampling.

Exact sample sizes are indicated in the figure legends.

Data exclusions

In vivo experiments: Significant outliers identified by ROUT method with Q = 1% were excluded, which applied to the experiments described in Fig. 3c and Fig. 5g. The excluded data can be indicated in a Source Data File. In Fig. 2e, amounts of F-actin in upstream and downstream injured ISVs during their repair processes were quantified. For that, fluorescence intensity of Lifeact-mCherry in regions of vessel branches within 20 µm from the leading edge was measured. In some experiments, leading edges regions were overlapped with intense autofluorescence derived from the debris generated during laser ablation, preventing accurate measurement of fluorescence intensity of Lifeact-mCherry. Thus, those data were excluded from analysis as described in a Source Data File.

In vitro experiments: No data were excluded.

Replication

In vivo experiments: At least four and two independent experiments were performed to confirm the reproducibility of the findings for quantitative and qualitative analyses, respectively. All replication attempts were successful.

In vitro experiments: All experiments for on-chip angiogenesis assay were repeated at least three times to secure reproducibility except for localization analysis of exogeneous EGFP-ARPC4 in stalk cells in Supplementary Fig. 10f, g. Replicated experiments were successful.
To investigate the effect of loading of intraluminal pressure on leading edge localization of Arp2/3 complexes in endothelial stalk cells (Supplementary Fig. 10f, g), 9 and 7 stalk cells expressing EGFP-ARPC4 in angiogenic branches loaded without (Control) or with intraluminal pressure were analyzed in one experiment, respectively. As a result, 8 out of 9 stalk cells in control angiogenic branches exhibited leading edge localization of EGFP-ARPC4, while none of analyzed stalk cells in the branched loaded with intraluminal pressure (7 stalk cells) did not.

These results suggest that loading of intraluminal pressure into angiogenic branches inhibits leading edge localization of Arp2/3 complexes in the endothelial stalk cells.
For in vitro wound healing assay, two independent experiments were performed. Replicated experiments were successful.

Randomization

In vivo experiments:
Adult and larval zebrafish were randomly selected for the experiments to analyze repair processes of injured blood vessels and hemodynamics in blood vessels.

For the experiments to analyze amount of F-actin, front-rear endothelial cell polarity, and localization of EGFP-ARPC4 and EGFP-Toca1 in injured intersegmental vessels, zebrafish larvae were randomly selected from those exhibiting moderate fluorescent signal of reporter proteins, because it is hard to precisely analyze localization of reporter proteins if the larvae showed weak fluorescent signal. Before the experiments, we confirmed that expression of reporter proteins did not affect formation and morphology of intersegmental vessels. Thus, selection of zebrafish larvae showing moderate fluorescence signal allowed us to analyze localization of reporter proteins precisely.

Littermates were used for analysis of toca1 and cip4 mutant zebrafish.

In Supplementary Fig. 12e, f, we investigated whether blood flow-mediated endothelial cell polarity affected elongation of upstream injured vessels. For this purpose, we examined elongation of upstream and downstream injured vessels in the larvae that exhibited defective blood flow-mediated endothelial cell polarization. Therefore, the larvae in which endothelial cells in upstream injured vessels positioned their Golgi apparatus in front or middle of the nucleus toward the vessel elongation direction immediately after injury were selected for analysis.

In vitro experiments:
In on-chip angiogenesis assays, all cultured cells were randomly allocated into different experimental groups in individual assays. For quantification analysis, to limit bias, data were collected from all branches or cells observed in each chip or randomly selected branches in each chip and the collection was repeated at least three times.

For in vitro wound healing assay, at least 6 regions per culture sample were randomly imaged for each experimental group.

Blinding

In vivo experiments: For the analysis of zebrafish mutants, wild type, heterozygous, and homozygous toca1 and cip4 mutants were imaged and analyzed blindly, because their genotyping was performed after the analysis. For other in vivo experiments, blinding during data collection was not performed since the same investigator did all experimental processes from group allocation to data analysis.

In vitro experiments: In all on-chip angiogenesis assays and in vitro wound healing assays, blinding during data collection was not performed since the same investigator did all experimental processes from group allocation to data analysis.

Reporting for specific materials, systems and methods

We require information from authors about some types of materials, experimental systems and methods used in many studies. Here, indicate whether each material, system or method listed is relevant to your study. If you are not sure if a list item applies to your research, read the appropriate section before selecting a response.

Materials & experimental systems

n/a	Involved in the study
☐	☒ Antibodies
☐	☒ Eukaryotic cell lines
☒	☐ Palaeontology and archaeology
☐	☒ Animals and other organisms
☒	☐ Human research participants
☒	☐ Clinical data
☒	☐ Dual use research of concern

Methods

n/a	Involved in the study
☒	☐ ChIP-seq
☒	☐ Flow cytometry
☒	☐ MRI-based neuroimaging

Antibodies

Antibodies used

Analysis of hypoxia in in vivo imaging - Primary: anti-GFP rabbit pAb (Thermo Fisher, A-11122, 1:500), anti-pimonidazole mouse mAb (C4.3.11.3, Hypoxiprobe, HP1, 1:50); Secondary: Alexa Fluor 488-labeled goat anti-rabbit IgG Ab (Thermo Fisher, A11034, 1:500), Alexa Fluor 546-labeled goat anti-mouse IgG Ab (Thermo Fisher, A11030, 1:500)

Western Blot - Primary: rabbit anti-CIP4 pAb (Proteintech, 10798-1-AP, 1:1,000), rabbit anti-GAPDH mAb (14C10, Cell Signaling, 2118, 1:2,000), anti-GFP rabbit pAb (Thermo Fisher, A-11122, 1:2,000), anti-actin mouse mAb (C4, BD Biosciences, 612656, 1:3,000); Secondary: IRDye 800CW goat anti-rabbit IgG Ab (LI-COR, 925-32211, 1:10,000), HRP-linked goat anti-rabbit IgG Ab (Cell signaling, 7074, 1:5,000), HRP-linked donkey anti-rabbit IgG Ab (GE Healthcare, NA934, 1:3,000), HRP-linked sheep anti-mouse IgG Ab (GE Healthcare, NA931, 1:3,000)

Immunofluorescence staining in on-chip angiogenesis assay - Primary: rabbit anti-VE-cadherin (D87F2) mAb (Cell Signaling, 2500, 1:500), mouse anti-CD31 (WM59) mAb (BioLegend, 303102, 1:500), rabbit anti-GOLPH4 mAb (Abcam, ab28049, 1:500), rabbit anti-p34-Arc/ARPC2 pAb (Merck, 07-227, 1:500), goat anti-ARPC2 pAb (Novus Biologicals, NB100-1037, 1:500), rabbit anti-CIP4 pAb (Proteintech, 10798-1-AP, 1:500), rabbit anti-N-WASP (30D10) (Cell Signaling, 4848, 1:100); Secondary: Alexa Fluor 488-labeled goat anti-rabbit IgG Ab (Thermo Fisher, A11034, 1:1,000) , Cy3-labeled goat anti-rabbit IgG Ab (Thermo Fisher, A10520, 1:1,000), Alexa Fluor 633-labeled goat anti-rabbit IgG Ab (Thermo Fisher, A21070, 1:1,000), Alexa Fluor 555-labeled goat anti-mouse IgG Ab (Thermo Fisher, A28180, 1,1,000), Alexa Fluor 488-labeled donkey anti-goat IgG Ab (Thermo Fisher, A-11055, 1,1,000), Alexa Fluor 555-labeled

donkey anti-goat IgG Ab (Thermo Fisher, A21432, 1,1,000),

In vitro wound healing assay - Primary: rabbit anti-p34-Arc/ARPC2 pAb (Merck, 07-227, 1:500), rabbit anti-CIP4 pAb (Proteintech, 10798-1-AP, 1:750), anti-cdh5 mAb (75, BD, 610251, 1:300); Secondary: Alexa Fluor 488-labeled goat anti-rabbit IgG Ab (Thermo Fisher, A11034, 1:500), Alexa Fluor 633-labeled goat anti-mouse IgG Ab (Thermo Fisher, A21052, 1:500)

Whole-mount in situ hybridization: sheep anti-Digoxigenin-AP, Fab fragments (Roche, 11093274910, 1:1,000)

Validation

All the antibodies used in this study were commercially available and validated by manufacturers as described on the following web sites or by previous reports.

Rabbit anti-GFP pAb (Thermo Fisher, A-11122): https://www.thermofisher.com/antibody/product/GFP-Antibody-Polyclonal/A-11122

Mouse anti-pimonidazole mAb (Hypoxiprobe, HP1): Shimada et al. Sci. Rep. 2018;8:16808.

Rabbit anti-CIP4 pAb (Proteintech, 10798-1-AP): https://www.ptglab.com/products/TRIP10-Antibody-10798-1-AP.htm

Rabbit anti-GAPDH mAb (Cell Signaling, 2118): https://en.cellsignal.jp/products/primary-antibodies/gapdh-14c10-rabbit-mab/2118

Mouse anti-actin mAb (BD Biosciences, 612656): https://www.bdbiosciences.com/en-us/products/reagents/microscopy-imaging-reagents/immunofluorescence-reagents/purified-mouse-anti-actin-ab-5.612656

Rabbit anti-VE-cadherin (D87F2) mAb (Cell Signaling, 2500): https://en.cellsignal.jp/products/primary-antibodies/ve-cadherin-d87f2-xp-rabbit-mab/2500

Mouse anti-CD31 (WM59) mAb (BioLegend, 303106): https://www.biolegend.com/ja-jp/products/purified-anti-human-cd31-antibody-883?GroupID=BLG5721

Rabbit anti-GOLPH4 mAb (Abcam, ab28049): https://www.citeab.com/antibodies/734019-ab28049-anti-golph4-antibody

Rabbit anti-p34-Arc/ARPC2 pAb (Merck, 07-227): https://www.emdmillipore.com/US/en/product/Anti-p34-Arc-ARPC2,MM_NF-07-227-I-100UG?bd=1

Goat anti-ARPC2 pAb (Novus Biologicals, NB100-1037): https://www.novusbio.com/products/arpc2-antibody_nb100-1037

Rabbit anti-N-WASP (30D10) (Cell Signaling, 4848): https://en.cellsignal.jp/products/primary-antibodies/n-wasp-30d10-rabbit-mab/4848?Ns=productCitationsCount%7C1&N=102236+3241918245+4294956287&Nrpp=1000&No=%7Boffset%7D&fromPage=plp

Mouse anti-Cdh5 mAb (BD, 610251): https://www.bdbiosciences.com/en-us/products/reagents/microscopy-imaging-reagents/immunofluorescence-reagents/purified-mouse-anti-cadherin-5.610251. Ando et al. J. Cell Biol. 202: 901-916, 2013.

Eukaryotic cell lines

Policy information about cell lines

Cell line source(s)

For on-chip angiogenesis assay: HUVECs and hLFs were purchased from Lonza. RFP-HUVECs were purchased from Anigio-Proteomie.
For in vitro wound healing assay: HUVECs were purchased from KURABO.

Authentication

Authentication was conducted by each manufacturer.

Mycoplasma contamination

All these cell lines were tested mycoplasma-negative by each manufacturer.

Commonly misidentified lines
(See ICLAC register)

No commonly misidentified cell lines were used in this study.

Animals and other organisms

Policy information about studies involving animals; ARRIVE guidelines recommended for reporting animal research

Laboratory animals

Zebrafish of the AB strain (Danio rerio) were used in this study. Adult male zebrafish at 3-18 months post-fertilization and larval zebrafish at 3 days post-fertilization were used for the experiments.

Transgenic zebrafish lines used were as follows:

Tg(fli1a:EYFP-Golgi)ncv510Tg, Tg(fli1a:EGFP-ARPC4)nf5Tg, and Tg(fli1a:EGFP-toca1)nf10Tg lines were generated in this study as described in the Methods section.

Tg(kdrl:EGFP)s843 and Tg(fli1a:EGFP)y1 lines were provided by D. Y. Stainier (Max Planck Institute for Heart and Lung Research) and N. Lawson (University of Massachusetts Medical School), respectively.

Tg(fli1a:Myr-EGFP)ncv2Tg, Tg(flt1enh:mCherry)ncv30Tg, Tg(fli1a:Lifeact-mCherry)ncv7Tg, Tg(fli1a:mCherry)ncv501, TgBAC(pdgfrb:eGFP)ncv23Tg and Tg(fli1a:h2b-mCherry)ncv31Tg lines have previously been reported as described in the Methods section.

	toca1nf4/nf4 and cip4nf4/nf4 zebrafish mutants were generated by CRISPR/Cas9 technology in this study, as described in the Methods section.
Wild animals	No wild animals were used in this study.
Field-collected samples	No field-collected samples were used in this study.
Ethics oversight	Animal experiments were approved by the animal committees of the National Cerebral and Cardiovascular Center (#15010) and the Nippon Medical School (#28-010, #2020-054) and performed by following the guidelines of the National Cerebral and Cardiovascular Center and the Nippon Medical School.

Note that full information on the approval of the study protocol must also be provided in the manuscript.
